# Impact of hypoxia on chemoresistance of mesothelioma mediated by the proton-coupled folate transporter, and preclinical activity of new anti-LDH-A compounds

**DOI:** 10.1038/s41416-020-0912-9

**Published:** 2020-06-04

**Authors:** Giovanna Li Petri, Btissame El Hassouni, Rocco Sciarrillo, Niccola Funel, Giulia Mantini, Eveline A. Zeeuw van der Laan, Stella Cascioferro, Amir Avan, Paolo Andrea Zucali, Nadia Zaffaroni, Tonny Lagerweij, Barbara Parrino, Kees Smid, Marcello Deraco, Carlotta Granchi, Alicja Braczko, Ryszard T. Smolenski, Larry H. Matherly, Gerrit Jansen, Yehuda G. Assaraf, Patrizia Diana, Jacqueline Cloos, Godefridus J. Peters, Filippo Minutolo, Elisa Giovannetti

**Affiliations:** 1 Department of Medical Oncology, Cancer Center Amsterdam, VU University Medical Center, Amsterdam, The Netherlands; 2 Dipartimento di Scienze e Tecnologie Biologiche Chimiche e Farmaceutiche (STEBICEF), Università degli Studi di Palermo, Palermo, Italy; 3 Department of Hematology, Cancer Center Amsterdam, VU University Medical Center, Amsterdam, The Netherlands; 4 Department of Pediatric Oncology, Cancer Center Amsterdam, VU University Medical Center, Amsterdam, The Netherlands; 5 Azienda Ospedaliera Universitaria Pisana, Pisa, Italy; 6 Cancer Pharmacology Lab, Fondazione Pisana per la Scienza, Pisa, Italy; 7 Metabolic syndrome Research center, Mashhad University of Medical Sciences, Mashhad, Iran; 8 Department of Oncology, Humanitas Clinical and Research Center, IRCCS, Rozzano (Milan), Italy; 9 Molecular Pharmacology Unit, Fondazione IRCCS Istituto Nazionale dei Tumori, Milano, Italy; 10 Department of Neurosurgery, Neuro-Oncology Research Group, Cancer Center Amsterdam, VU University Medical Center, Amsterdam, The Netherlands; 11 Peritoneal Malignancy Program, Fondazione IRCCS Istituto Nazionale dei Tumori, Milano, Italy; 12 Department of Pharmacy, University of Pisa, Pisa, Italy; 13 Department of Biochemistry, Medical University of Gdansk, Gdańsk, Poland; 14 Barbara Ann Karmanos Cancer Institute, Wayne State University School of Medicine, Detroit, MI USA; 15 Amsterdam Rheumatology and immunology Center, VU University Medical Center, Amsterdam, The Netherlands; 16 Fred Wyszkowski Cancer Research Laboratory, Department of Biology, Technion-Israel Institute of Technology, Haifa, Israel

**Keywords:** Mesothelioma, Translational research

## Abstract

**Background:**

Expression of proton-coupled folate transporter (PCFT) is associated with survival of mesothelioma patients treated with pemetrexed, and is reduced by hypoxia, prompting studies to elucidate their correlation.

**Methods:**

Modulation of glycolytic gene expression was evaluated by PCR arrays in tumour cells and primary cultures growing under hypoxia, in spheroids and after PCFT silencing. Inhibitors of lactate dehydrogenase (LDH-A) were tested in vitro and in vivo. LDH-A expression was determined in tissue microarrays of radically resected malignant pleural mesothelioma (MPM, *N* = 33) and diffuse peritoneal mesothelioma (DMPM, *N* = 56) patients.

**Results:**

Overexpression of hypoxia marker CAIX was associated with low PCFT expression and decreased MPM cell growth inhibition by pemetrexed. Through integration of PCR arrays in hypoxic cells and spheroids and following PCFT silencing, we identified the upregulation of LDH-A, which correlated with shorter survival of MPM and DMPM patients. Novel LDH-A inhibitors enhanced spheroid disintegration and displayed synergistic effects with pemetrexed in MPM and gemcitabine in DMPM cells. Studies with bioluminescent hypoxic orthotopic and subcutaneous DMPM athymic-mice models revealed the marked antitumour activity of the LDH-A inhibitor NHI-Glc-2, alone or combined with gemcitabine.

**Conclusions:**

This study provides novel insights into hypoxia/PCFT-dependent chemoresistance, unravelling the potential prognostic value of LDH-A, and demonstrating the preclinical activity of LDH-A inhibitors.

## Background

Malignant pleural mesothelioma (MPM) and diffuse malignant peritoneal mesothelioma (DMPM) are rare but aggressive tumours arising from mesothelial cells lining the pleural and peritoneal cavity, respectively. The incidence of these malignancies is associated with exposure to asbestos, and is increasing throughout the world, with a predicted peak in the next 15 years.^1^ Both MPM and DMPM are typically diagnosed at an advanced stage, and are extremely difficult to treat.

Systemic therapy is the only treatment option for the vast majority of MPM patients. The standard of care in the first-line treatment is a combination of platinum-based chemotherapy with the third-generation antifolate pemetrexed. This combination significantly improved the overall survival (OS, 12.1 vs. 9.3 months; *P* = 0.020), compared with cisplatin monotherapy.^2^ Patients who do not qualify to receive cisplatin-based chemotherapy, are treated with alternative chemotherapy including pemetrexed alone, or in combination with carboplatin.^3^ Systemic chemotherapy is also used for patients harbouring DMPM, when patients cannot undergo cytoreductive surgery followed by hyperthermic perioperative chemotherapy.^4^

Coupled with a better understanding of the molecular mechanisms underlying drug resistance or sensitivity, the introduction of biomarkers into the pathologic analysis of both MPM and DMPM should drive the individualisation of precision medicine and improve the outcome of these malignancies. A few retrospective studies suggested the predictive role of the primary target of the activity of pemetrexed, thymidylate synthase (TS),^5,6^ but further research on additional mechanisms of chemoresistance is warranted.^7,8^

The proton-coupled folate transporter (PCFT/SLC46A1) has recently emerged for its key role in folate and antifolate transport, which was demonstrated in various model systems, including MPM,^9^ and in several human tissues.^10^ Of note, the activities of the folate transporters reduced folate carrier (RFC/SLC19A1), and PCFT is significantly affected by the pH of the tumour microenvironment (TME). Whereas RFC displays optimal transport activity at physiological pH, PCFT-mediated transport is very low.^11^ The optimal extracellular pH for PCFT-dependent transport is 5.5, with considerable transport activity detectable at pH 6.5 or 6.8, depending upon the tumour size, type and distance from blood vessels. Therefore, PCFT may be the sole route of delivery of folates and antifolates in the acidic microenvironment of solid tumours. Furthermore, PCFT exhibits a high transport affinity for pemetrexed,^12^ and PCFT transfection increases its folate and antifolate transport, as well as antifolate cytotoxicity.^11^ These findings support the unique role that PCFT plays in the transport and pharmacologic activity of pemetrexed; consistently, we recently demonstrated that low expression of the PCFT transporter, both at the mRNA and protein levels, is associated with shorter survival of MPM patients treated with pemetrexed.^13^ This enhanced our interest into additional key mechanisms associated with drug resistance underlying the modulation of PCFT expression in cancer cells. Interestingly, Raz and collaborators^14^ showed that severe hypoxia induced a complete antifolate resistance and caused simultaneous suppression of key genes in folate homoeostasis, including the influx transporters RFC and PCFT. This could be attributed, at least in part, to alterations in Sp1 activity or promoter methylation of these genes under hypoxic conditions. These data are in agreement with previous findings demonstrating that cells growing in three-dimensional (3D) systems, with increased hypoxic areas, showed diminished antifolate transport and decreased antifolate sensitivity.^15^ The current study was aimed at evaluating the correlation between PCFT expression under hypoxic conditions with pemetrexed activity in MPM cells. Moreover, using a variety of in vitro models, including 3D spheroids, as well as targeted silencing and PCR arrays, we sought to further elucidate key factors affecting drug resistance. These studies identified a significant upregulation of the key glycolytic enzyme LDH-A. Our previous studies demonstrated that novel LDH-A inhibitors were especially effective and synergistic with gemcitabine against cells under hypoxic conditions, and gemcitabine displayed antitumour activity in both MPM and DMPM patients.^16–20^ Moving from these premises, we investigated the role of LDH-A inhibition as a potential therapeutic strategy both in MPM and DMPM, using in vitro and in vivo orthotopic and subcutaneous models. Finally, we investigated whether the expression levels of LDH-A were associated with significantly worse clinical outcome, using tissue microarrays (TMA) with specimens from both MPM and DMPM patients. Our results provide novel mechanistic insights into mesothelioma chemoresistance that may contribute to the rational development of innovative prognostic biomarkers and therapeutic interventions for this devastating disease.

## Methods

### Patients and immunohistochemistry

Previous studies demonstrated that MPM is a hypoxic malignancy,^21,22^ and cells that survive hypoxia are more resistant to antifolates because of the downregulation of several key enzymes and transporters.^14^ Therefore, we evaluated by immunohistochemistry (IHC) the percentage and distribution of hypoxic cells by using the monoclonal antibody ab15086 (Abcam, Cambridge, MA) to detect the levels of carbonic anhydrase IX (CAIX), as described previously,^23^ within areas with high and low PCFT levels that were assessed with PCFT polyclonal antibody.^13^

The expression levels of these proteins were evaluated in tissue microarrays (TMA), including tumours from 33 MPM patients enrolled at Humanitas Cancer Center (Milan, Italy) between 2008 and 2013, as described previously.^13^ Further IHC studies were performed in order to assess the expression levels of LDH-A using both the TMA of MPM patient specimens and TMAs containing samples from 56 DMPM patients enrolled between 1995 and 2013 at the National Cancer Institute (INT, Milan, Italy), as described previously.^24^ Patients’ characteristics are summarised in Supplementary Table 1. These studies were approved by the appropriate ethical review boards (Humanitas Cancer Center (ClinicalTrials.gov NCT00867711) and INT Review Board and Ethics Committees). Scoring for PCFT was described in our previous study.^13^ Immunostaining intensity of CAIX was described in Supplementary Table 2, while for LDH-A, we used a previously proposed grading system with two LDH-A expression levels: strong cytoplasmic expression in >50% of cancer cells or nuclear expression in >10% of cancer cells was defined as high expression; otherwise, nuclear expression was considered low.^25^ More details on TMA and IHC are reported in the Supplemental Methods section.

### Cells and drugs

Three human MPM cell lines (H28, H2452 and MSTO-211H) were obtained from ATCC (Manassas, VA) and cultured as previously described.^26^ Human primary DMPM cultures (MesoII and STO) were derived from tumour samples of patients who underwent surgery, and were maintained in DMEM/F12 (Gibco) under standard culture conditions for less than 20 passages.^24^ Cells were routinely tested for mycoplasma. Analyses of mitochondrial function and glycolysis of MSTO-211H and H2452 cells were performed with the Seahorse XFp Metabolic Flux Analyzer (Agilent Technologies, Santa Clara, CA), showing that these cells have both a normal aerobic metabolism and aerobic glycolysis, though to a different extent, as reported in the Supplemental Methods and Supplementary Fig. S1.

Co-transduction of MesoII cells with luciferase vectors and Firefly-luciferase (F-luc) activity assessment were performed according to previously established methods.^23,24^ Pemetrexed and gemcitabine were gifts from Eli Lilly (Indianapolis, IN), while the LDH-A inhibitors NHI-2 and NHI-Glc-2 were synthesised as described previously.^17,27^ Drugs were dissolved in sterile water (gemcitabine) or dimethyl sulfoxide (DMSO, pemetrexed and LDH-A inhibitors) and diluted in culture medium immediately before use. In each experiment, we did not use concentrations higher than 0.1% DMSO.

### Evaluation of the role of hypoxia on PCFT expression and pemetrexed activity

The impact of hypoxia on the sensitivity of MPM cells to pemetrexed was evaluated by growing cells at an O_2_ tension of 1%, 5% (vol/vol) CO_2_ and 94% (vol/vol) N_2_ at 37 °C, using a specific IncuSafe Jacomex Glove Box (Labo Equipment Sanyo, Loughborough, UK). The concentrations of pemetrexed that inhibited cell growth by 50% (IC_50_) were determined using the sulforhodamine-B (SRB) assay, as described previously.^24^ Cells exposed for 72 h to hypoxic conditions were also used to explore the downregulation of PCFT expression by quantitative real-time PCR (qRT-PCR), as well as after silencing with the specific anti-PCFT siRNA D#141241-siRNA (Thermofisher, Waltham, MA). The PCR reactions were performed using the Hs00560565_m1 Assay-on-Demand product (Applied Biosystems, Foster City, CA), with the ABI PRISM^TM^ 3100 Genetic analyzer (Applied Biosystems). Further studies with qRT-PCR assessed the expression of LDH-A in MSTO and STO cells, using previously validated methods.^17^ In addition, the influence of PCFT on pemetrexed-mediated cell growth inhibition was studied in parallel SRB experiments after 48 h of exposure to the anti-PCFT siRNA or its negative control.

Finally, since previous studies showed that 3D MPM models have hypoxic cores, and are generally more chemoresistant than two-dimensional monolayer cell cultures,^17,24,28^ we performed additional experiments using spheroids that were also used for western blot analysis of LDH-A levels, as well as an exploratory analysis using a sequential trypsin digestion of spheroids of H2452 cells, as reported in the Supplemental Methods.

### Analysis of pathways involved in pemetrexed resistance after PCFT silencing and under hypoxic conditions

The molecular events occurring after PCFT silencing and exposure to hypoxic conditions in cells growing as monolayers, as well as spheroids, were evaluated using Hypoxia RT2 Profiler^TM^ PCR Arrays (Qiagen, Hilden, Germany), according to the manufacturers’ protocol. This array includes 84 key components of the molecular machinery that modulate cell metabolism in response to hypoxic signals. For this analysis, we used H2452 cells growing as monolayers treated for 48 h with the PCFT-specific siRNA, as well as cells exposed for 72 h to hypoxic conditions, and spheroids, as described above.

### Pharmacological interaction of NHI-2 and NHI-Glc-2 with pemetrexed and gemcitabine

The cell growth-inhibitory effects of the combination of the LDH-A inhibitors NHI-2 and NHI-Glc-2 and pemetrexed were evaluated in spheroids of H2452 cells. These spheroids were treated simultaneously with 1 µM NHI-2 and 1 μM pemetrexed for 7 days. The cytotoxic effects were evaluated by determining the density and size of spheroids, as described previously.^17,24^ Then, we evaluated the induction of apoptosis in H2452 cells growing as monolayers under hypoxic conditions and treated with 1 µM NHI-2 alone and 1 μM pemetrexed for 24 h. The apoptotic index was calculated after bisbenzimide–HCl staining, as described previously.^29^ Further studies evaluated the pharmacological interaction of NHI-Glc-2 with gemcitabine using cells growing either in monolayers, under normoxic and hypoxic conditions, or as spheroids.

### In vivo experiments using orthotopic and subcutaneous mouse models and live imaging

In vivo experiments were performed in nu/nu athymic female mice purchased from Harlan (Horst, The Netherlands). The working protocol was approved by the local committees on animal experimentation of the VU University Medical Center (VUmc, Amsterdam, The Netherlands) and of the University of Pisa (Pisa, Italy), according to the 2010/63/EU European Community Council Directive for laboratory animal care.

Orthotopic primary DMPM models (*n* = 5 tumours per treatment group) were generated by injection of 3 × 10^6^ Fm/GC primary cells into the peritoneal cavity of the mice. Mice were treated with NHI-Glc-2, solubilised in Polyethylene glycol 400 (PEG400, Sigma-Aldrich, St. Louis, MO), at 100 mg/kg, 5 days (1–5) for 2 weeks (formulation concentration: 25 mg/mL in PEG400, 100 µL of i.p. injection for a 25-g mouse). Bioluminescence imaging (BLI) was evaluated with a Bruker In-Vivo Xtreme Capture System, using Molecular Imaging Software (Bruker Corporation, Billerica, MA). Additional imaging analyses to define tumour spatial characteristics and evaluate microenvironment structures, such as neovasculature and hypoxic status, were carried out by high-frequency ultrasound including Power Doppler Mode (Vevo-2100, VisualSonics, Amsterdam, The Netherlands). Data normalisation and image analysis were performed as described previously.^23,24^

Further experiments were performed on subcutaneous tumours, obtained by inoculation of 3 × 10^6^ tumour cells. In these models, we also tested drug combinations. Since (1) pemetrexed activity cannot be reliably evaluated in mouse models, because of the intrinsically high levels of folate and thymidine,^30^ and (2) our previous experiments showed a synergistic interaction of LDH-A inhibitors with gemcitabine, we used LDH-A inhibitors in combination with gemcitabine.^17^

When tumour volume reached an average size of 100 mm^3^, the animals were randomly distributed into four groups (*n* = 6 tumours per treatment group) as follows: (1) control/untreated mice, (2) mice treated with gemcitabine alone at 100 mg/kg, 2 days (days 1 and 4) for 3 weeks (formulation concentration: 25 mg/mL in PBS, 100 µL of i.p. injection for a 25-g mouse), (3) mice treated with NHI-Glc-2, solubilised in PEG400, at 50 mg/kg, 5 days (days 1–5) for 3 weeks (formulation concentration: 12.5 mg/mL in PEG400) and (4) mice treated with a simultaneous combination of gemcitabine and NHI-Glc-2, at the doses mentioned above, for 3 weeks. Tumour xenografts were measured as described previously.^31^ Lidocaine was used as local anaesthetic on the skin of the mice. Mice were sacrificed before primary tumours and metastases can cause severe symptoms, via cervical dislocation.

### Statistics

Clinical outcome was correlated with demographic/clinicopathological information parameters, and CAIX and LDH-A expression by univariate analysis using the Chi-square test. We modified RECIST criteria to classify the MPM response to treatment as complete response (CR), partial response (PR), stable disease (SD) or progressive disease (PD). The patients who showed disease control (DC), including CR, PR and SD, were compared with patients with PD, as described previously.^6^ Survival curves (OS and progression-free survival, PFS) were analysed from the day of initiation of drug treatment to the endpoint (death or censoring) according to the Kaplan–Meier method, and compared by log-rank and Wilcoxon tests, using SPSS software Version 24 (IBM-SPSS, Chicago, IL). Significant prognostic variables identified by univariate analysis were included in the multivariate analysis, using Cox’s proportional hazard model and the backward stepwise elimination (Wald) method, where hazard ratios (HRs) were calculated to estimate the magnitude and the direction of the effect. The in vitro experiments were performed in triplicates and repeated at least twice. The results reported in the figures are expressed as mean values ± standard error of the mean (SEM). Statistical analyses were carried out by two-way ANOVA followed by Bonferroni’s post-test (to adjust for multiple comparisons), using GraphPad-Prism version 7 (Intuitive Software for Science, San Diego, CA). All analyses were two-sided, and statistical significance was set at *P* < 0.05. The in vivo experiments included as the primary and secondary experimental outcomes the assessment of tumour growth and of molecular markers (pO_2_ and LDH-A). Statistical analysis was conducted with ANOVA analysis with Bonferroni correction for post hoc analysis to evaluate differences in tumour size in the different groups of animals. *P* < 0.05 was considered significant. The results were expressed as mean values ± standard deviation (SD).

## Results

### Role of hypoxia in modulation of PCFT expression and pemetrexed cytotoxicity

**Fig. 1:**
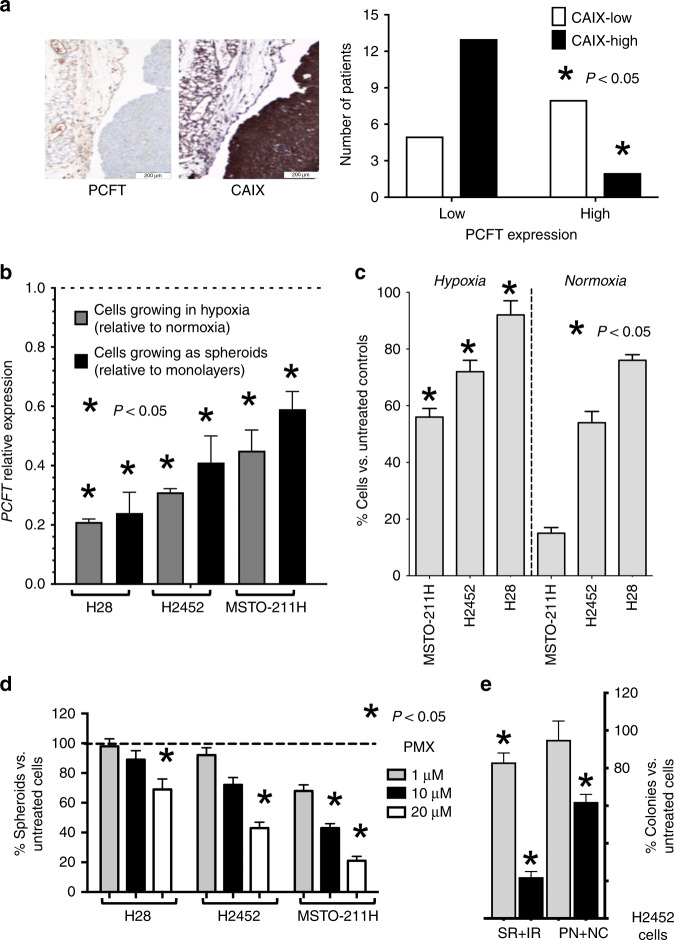
Hypoxia affects PCFT expression and pemetrexed activity. **a** Left panel: representative immunohistochemical pictures of two consecutive sections of an MPM tissue stained with anti-PCFT and anti-CAIX antibodies (left and right pictures, respectively, original magnification ×20). Right panel: analysis of the inverse/negative correlation of PCFT and CAIX staining in MPM patients (*N* = 28). **b** Quantitative RT-PCR analysis of PCFT mRNA expression in human MPM cell lines growing under hypoxic conditions or as spheroids. For each mesothelioma cell line, the results are presented relative to the expression levels of PCFT in cells growing in normoxia as two-dimensional monolayer cell cultures, assigned a value of 1 (dashed line). *Columns*, mean values obtained from three independent experiments; *bars*, SEM. *Significantly different (*P* < 0.05) compared with the untreated cells under normoxic conditions. **c** Cell growth inhibition performed with cells exposed to 1 µM pemetrexed (PMX) for 72 h under hypoxic vs. normoxic conditions, as compared with drug-free control cells. *Columns*, mean values obtained from three independent experiments; *bars*, SEM. *Significantly different (*P* < 0.05) compared with the same treatments under normoxic conditions. **d** Relative number of spheroids originating from H2452 cells treated with PMX compared with spheroids originating from drug-free cells, assigned a value of 100%. *Columns*, mean values obtained from three independent experiments; *bars*, SEM. *Significantly different (*P* < 0.05) compared with spheroids growing from untreated cells. **e** Relative number of colonies originated from the perinecrotic region and necrotic core (PN + NC) compared with the surface and intermediate regions (SR + IR) of H2452 spheroids treated with 1 or 10 µM PMX for 72 h, compared with spheroids originating from untreated cells, assigned as a value of 100%. *Columns*, mean values obtained from three independent experiments; *bars*, SEM. *Significantly different (*P* < 0.05) compared with untreated cells.

Since previous studies showed that hypoxia induces chemoresistance in different tumour types including MPM,^22,32^ we evaluated the hypoxia marker CAIX in the TMA of primary MPM specimens. Most samples (84%; 28 out of 33) showed positive staining for this hypoxia marker, as previously reported.^21^ Remarkably, a stronger and diffuse staining of CAIX was typically associated with reduced PCFT expression (Fig. 1a). These findings are in agreement with previous results in carcinoma cells exposed to severe hypoxic conditions.^14^ When the tissues positive for CAIX staining were grouped into high- and low expression according to the scoring system reported in Supplementary Table 2, we observed that high expression levels of CAIX were associated with low expression of PCFT (*P* = 0.016, Fig. 1a), which was assessed as described previously.^18^ These results are in agreement with the role of this folate/antifolate transporter in cells with acidic pH,^33^ which commonly characterises the TME. To further explore the molecular mechanisms underlying the role of hypoxia in the modulation of PCFT and chemoresistance, we then evaluated PCFT expression in MPM cells cultured under hypoxic conditions. As shown in Fig. 1b, PCFT expression was downregulated under hypoxia in all the three MPM cell lines examined. This was accompanied by increased resistance to pemetrexed, compared with its counterparts grown under normoxia, with a significant inhibition of cell growth (as assessed by direct viable cell counting) upon exposure to 0.1, 1 and 10 µM pemetrexed (Fig. 1c; Supplementary Fig. S2). A similar reduction of PCFT expression was also detected in the MPM spheroids (Fig. 1b), and pemetrexed significantly affected the number of spheroids only at high concentrations, up to 1, 10 and 20 µM, in MSTO-211H, H2452 and H28 cells, respectively (Fig. 1d). We hypothesised that the reduction of PCFT expression was correlated to the hypoxic regions close to the core of these spheroids. Indeed, using a sequential trypsin digestion of spheroids of H2452 cells that had reached a diameter of approximately 500 μm, we observed that cells from the perinecrotic region and necrotic core (PN + NC) were considerably less sensitive to drug activity compared with cells from the surface and intermediate regions (SR + IR), as reported in Fig. 1e.

### Role of LDH-A in the modulation of PCFT expression

**Fig. 2:**
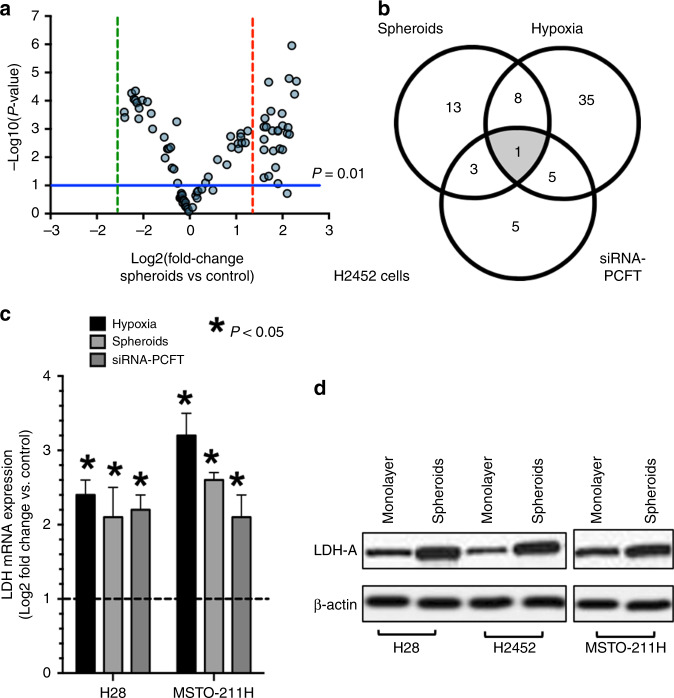
Correlation of hypoxia and PCFT silencing with LDH-A expression. **a** Volcano plot depicting the results of PCR arrays in H2452 spheroids compared with H2452 cells growing as monolayers. The horizontal blue line identifies the cut-off for genes that displayed a significantly different expression (*P* values were calculated with the two-sided Student's *t* test); vertical red and green lines mark the cut-offs for the genes with threefold up- or downregulation, respectively. **b** Venn diagram of the overlap analysis of genes significantly upregulated in hypoxic cells, as well as in cells growing as spheroids, and after specific downregulation of PCFT expression in H2452 cells. **c** Modulation of LDH-A expression in hypoxia, spheroids and after PCFT silencing. MPM cell lines showed a significant modulation of LDH-A mRNA expression when growing under hypoxic conditions, as spheroids or after PCFT silencing. *Columns*, mean values obtained from three independent experiments; *bars*, SEM. *Significantly different (*P* < 0.05) from the respective control cells, i.e., cells growing in normoxia for the cells growing in hypoxia, cells growing as monolayers for the cells growing as spheroids and cells exposed to siRNA-negative control for the cells exposed to siRNA PCFT silencing, respectively, exemplified by the dashed line, with expression values of 1. **d** Representative immunoblots illustrating the modulation of LDH-A protein expression in MPM cells growing as spheroids compared with cells growing as monolayers.

To shed light on the molecular mechanisms underlying the chemoresistance of MPM cells to pemetrexed under hypoxia, as well as in cells growing as spheroids, and after specific downregulation of PCFT expression, we performed a PCR array focusing on key regulators of hypoxia response. The volcano plot depicted in Fig. 2a arranged the genes according to the extent of differential expression (either up- or downregulation, *X* axis) and statistical significance with *P* values <0.01 (*Y* axis) for both the up- or downregulated genes in the H2452 spheroids, compared with cells growing in monolayers. Setting a threefold change as the cut-off level, this analysis identified 25 significantly upregulated genes (Supplementary Table 3). These include the hypoxia-inducible factor-1 (*HIF-1*) and co-transcription factors *ARNT*, *HIF1A*, *HIF3A*, *HNF4A* and *NCOA1*, as well as genes involved in glycolytic metabolism, such as *LDH-A*, *ALDOA*, *ENO1*, *HK2*, *PDK1*, *PFKFB4*, *PGK1*, *PKM*, *SLC2A1* and *SLC2A3*, but also the co-regulators of apoptosis and cell proliferation *ADM*, *BTG1* and *PIM1*. We also detected a marked increase in *MET* levels, as well as of the pleiotropic transcription factor *NFKB1*, both of which are frequently overexpressed in MPM and can suppress pro-apoptotic signalling pathways, promoting malignant behaviour and chemoresistance.^34–36^ Finally, we observed a significant increase in *MMP9* levels, which has been linked to invasion/metastasis in several tumour types, including MPM.^37^

As depicted in the Venn diagram in Fig. 2b, only nine of these significantly upregulated genes in the H2452 spheroids were also among the 49 genes that were upregulated under hypoxia, and there were only four common genes (i.e., *HIF1A*, *LDH-A*, *NFKB1* and *MMP9*) after *PCFT* silencing (Supplementary Table 3). Interestingly, the only upregulated gene identified in all three conditions was *LDH-A*, which encodes for a key enzyme catalysing the conversion of l-lactate and NAD to pyruvate and NADH in the final step of anaerobic glycolysis. Using the same conditions (i.e., spheroids, hypoxia exposure and PCFT silencing), we also observed a significant upregulation of *LDH-A* in H28 and MSTO-211H cells (Fig. 2c). The online database STRING, which allows the retrieval of the functional and physical interactions of proteins, did not identify a physical interaction between PCFT and LDH-A, as shown in Supplementary Fig. S3A. The only common node, creating a network with low-stringency settings, was the ubiquitin C protein, which is involved in the post-transcriptional modification of both these target proteins. It is well known that hypoxia, through HIF-1α, triggers the upregulation of genes that are critical for the promotion of glycolysis, including LDH-A.^32^ In addition, three putative nuclear respiratory factor-1 (NRF-1)-binding sites have been identified in the PCFT promoter, and compelling evidence established NRF-1 as a major inducible transcriptional regulator of PCFT, thereby linking folate transport with mitochondria biogenesis and cell metabolism.^38^ However, the present study is the first to identify a relationship between PCFT silencing and LDH-A overexpression. One may hypothesise that low PCFT expression could stimulate the increased expression of LDH-A as a feedback mechanism (Supplementary Fig. S3B). The increase in LDH-A activity, which catalyses the reversible transformation of pyruvate into lactate, might determine an extracellular acidification due to the lactate secretion and, consequently, would favour folate absorption through PCFT. We hypothesise that this increased PCFT activity would counteract the reduced expression of PCFT in hypoxic conditions. Of note, we also observed that LDH-A protein was overexpressed in MPM spheroids from all three MPM cell lines, compared with attached monolayer cells (Fig. 2d). However, the western blot analyses of LDH-A in the spheroids were limited by the fact that we used the homogenates of the entire spheroids, and could not estimate the spatial distribution of LDH-A, which was presumably differentially expressed in the peripheral and core regions of these spheroids. Since these spheroids were fragile, we could not use our recently developed method with confocal microscopy,^39^ in order to evaluate the localisation of LDH-A in terms of expression at the surface compared with the hypoxic regions close to the necrotic core.

### Preclinical activity of anti-LDH-A compounds in MPM cell lines and DMPM primary cultures growing as monolayers or as spheroids

**Fig. 3:**
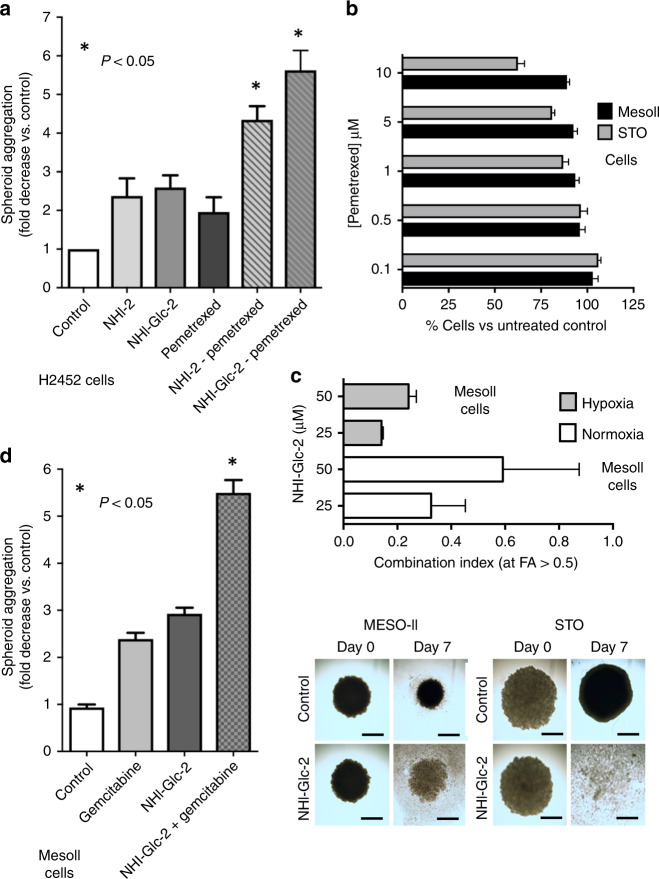
Cytotoxic effect of anti-LDH-A compounds alone and in combination with pemetrexed or gemcitabine on MPM and DMPM monolayers and spheroids. **a** Evaluation of the inhibition of the aggregation of the spheroids treated with pemetrexed (PMX), alone or in combination with 1 µM NHI-2, or NHI-Glc-2, compared with untreated spheroids originating from H2452 cells. The spheroids had similar volumes (below 500 µm^3^) at the start of drug exposure, and the untreated spheroids were still growing during the following 72 h. *Columns*, mean values obtained from three independent experiments; *bars*, SEM. *Significantly different (*P* < 0.05) from cells treated with PMX alone. **b** Cell viability bar graph of MesoII and STO cell lines treated with PMX. *Columns*, mean values obtained from three independent experiments; *bars*, SEM. **c** Combination index values, calculated at FA > 0.5, with Calcusyn software, as described in the “Methods” section. *Columns*, mean values obtained from three independent experiments; *bars*, SEM. **d** Evaluation of the inhibition of the aggregation of the spheroids treated with gemcitabine, alone or in combination with 10 µM NHI-Glc-2, compared with untreated spheroids originating from MesoII cells. *Columns*, mean values obtained from three independent experiments; *bars*, SEM. *Significantly different (*P* < 0.05) from cells treated with gemcitabine alone. Right panel: representative images of DMPM spheroids. The spheroids had similar volumes (below 500 µm^3^) and density at the start of drug exposure. However, after 7 days, the volume of untreated spheroids was reduced, while the density was significantly increased. In comparison with untreated spheroids (control), the treatment for 7 days with 25 µM of NHI-Glc-2 dramatically reduced the volume of STO spheroids, while the MesoII spheroids showed about a threefold decrease in their aggregation (considering both the volume and density, as explained in the “Methods” and Supplemental Methods and Supplementary Fig. S6). Scale bar, 100 µm. *Column*s, mean values obtained from three independent experiments; *bars*, SEM. *Significantly different (*P* < 0.05) from cells untreated.

To explore whether the increase in LDH-A expression is vital for cells with low PCFT levels, we treated spheroids of H2452 cells with the specific LDH-A inhibitor NHI-2, and assessed the antitumour effects by determining the number and density/size of the spheroids. The total number of spheroids was not affected, but treatment with NHI-2 substantially increased the disintegration of the spheroids. In particular, NHI-2 significantly reduced the size of spheroids, indicated as spheroid aggregation in Fig. 3a, compared with the drug-free control, and to the spheroids treated with pemetrexed alone. Moreover, NHI-2 enhanced both the pro-apoptotic (Supplementary Fig. S4A) and the cytotoxic effect of pemetrexed in these MPM models (Fig. 3a). Similar results were obtained with the glycoconjugated LDH-A inhibitor NHI-Glc-2 (Fig. 3a), which was synthesised to exploit the elevated glucose uptake of cancer cells, and showed increased intracellular concentration compared with NHI-2.^27^

Since early passages of primary mesothelioma cells may better mimic the genetic characteristics of the disease, we extended our studies to two primary DMPM cell cultures, MesoII and STO. However, the percentages of cell growth of these cells were not affected by treatment with pemetrexed at concentrations until 10 µM, as shown in Fig. 3b. This is in line with the lack of clinical activity of pemetrexed in DMPM. Thus, we did not perform further studies with pemetrexed in DMPM cells.

Since our previous study showed a synergistic interaction of LDH-A inhibitors with gemcitabine against pancreatic cancer cell lines in hypoxia,^17^ we explored the cytotoxic effects of NHI-Glc-2 in combination with gemcitabine on MesoII and STO cells, cultured under normoxic and hypoxic conditions (1% O_2_), as well as cells growing as adherent monolayers or spheroids. We initially treated cells growing under normoxic conditions with NHI-Glc-2 (0.5–50 µM), and observed a similar growth-inhibitory effect in both tumour cell lines (IC_50_ = 20.1 in MesoII and 18.8 µM in STO cells, respectively). We then investigated the pharmacological interaction of this LDH-A inhibitor with an IC_25_ concentration of gemcitabine (i.e., 5.5 nM in MesoII and 1.9 nM in STO) in cells growing under normoxic and hypoxic conditions (Supplementary Fig. S4B). In both primary cell cultures, the combination index values (CI) indicated synergistic or strong synergistic effects that were mainly evident at 25 µM of NHI-Glc-2 in MesoII cells (Fig. 3c).

Moreover, we evaluated the efficacy of this new combination on newly established 3D models of our primary cell cultures. Notably, in these models, we observed an increase in LDH-A expression over time as detected by qRT-PCR (Supplementary Fig. S5), hence recapitulating the findings observed under hypoxia in different cell tissues.^32^

The spheroids were treated with 10 and 25 µM of NHI-Glc-2 for 7 days, and we observed a significant reduction in density compared with untreated spheroids, as assessed with ImageJ (Supplementary Fig. S6). In particular, the combination with gemcitabine (10 nM) was additive when NHI-Glc-2 was applied at a concentration of 10 µM (Fig. 3d), while gemcitabine did not increase significantly the disaggregation of spheroids when NHI-Glc-2 was given at a concentration of 25 µM (data not shown). This is ostensibly due to the fact that this concentration would already greatly affect the structure of the spheroids, and could not further synergise with gemcitabine.

### Antitumour activity of NHI-Glc-2 in DMPM xenografts

We recently developed two novel bioluminescent (BLI) orthotopic mouse models in order to monitor tumour growth of primary DMPM cells MesoII and STO over time.^24^ The model with MesoII cells was selected for this study because of the higher level of LDH-A expression, as assessed by RNA sequencing, as described previously.^24^ Moreover, the analysis of our RNA sequencing data (raw and normalised data were deposited at Gene Expression Omnibus; accession number: GSE112154) showed a significantly higher expression of genes in the LDH-A family in DMPM tissues compared with normal mesothelial cells (Supplementary Fig. S7).

**Fig. 4:**
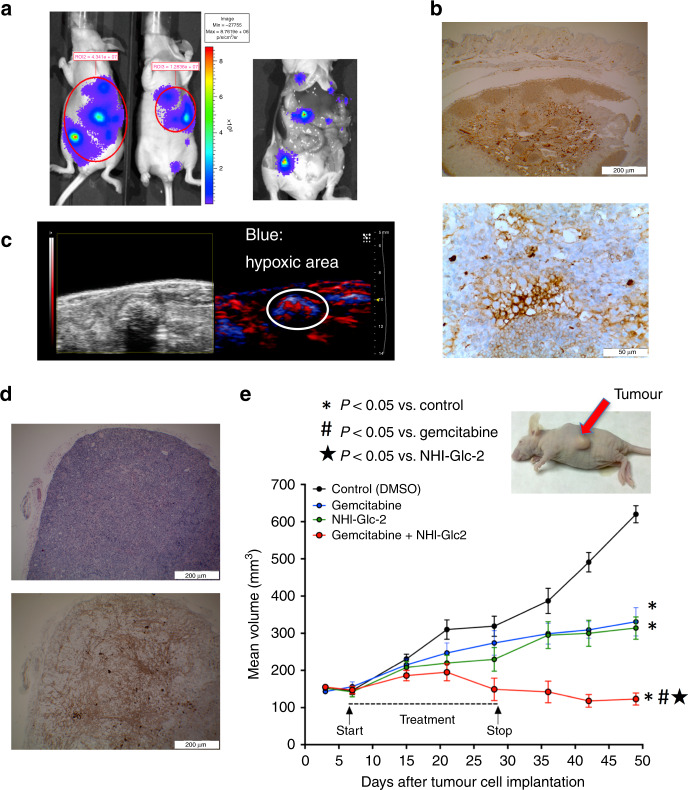
In vivo activity of the new anti-LDH-A compound NHI-Glc-2 on orthotopic and subcutaneous DMPM models. **a** Left panel: representative BLI images obtained with the CCD camera of orthotopic models of primary DMPM cells transduced with F-luc. Right panel: representative images showing the multiple tumour masses in the whole abdominal cavity as nodules, thus reproducing the diffusion pattern of the clinical disease in a mouse sacrificed after 2 weeks from inoculation of MesoII cells. **b** Representative immunohistochemical pictures (upper panel ×4, lower panel ×40 magnification) showing LDH-A overexpression in DMPM tissues obtained after orthotopic implantation of MesoII cells in mice. **c** Representative images of photoacoustic live imaging providing both a non-invasive anatomical image of tumours (up to 40-μm resolution) and the measurement of deep-tissue pO_2_. The latter showed reduced oxygenation (in blue), suggesting that tumour nodules were characterised by hypoxic regions. **d** Representative H&E (upper panel), and immunohistochemical (lower panel) pictures (×4 magnification) demonstrating LDH-A overexpression that characterised subcutaneous tumours obtained by inoculation of MesoII cells in mice. **e** Volumes of subcutaneous tumours of mice, as shown in the representative picture, treated with gemcitabine (100 mg/kg, i.p., 2 days a week), NHI-Glc-2 (solubilised in PEG400, 50 mg/kg, 5 days a week) or their combination compared with untreated mice. *Points*, mean values obtained from six mice; *bars*, SEM. *Significantly different (*P* < 0.05) from the untreated animal. # and ⋆ significantly different from animals treated with gemcitabine or NHI-Glc-2 monotherapy, respectively.

A rotational BLI analysis using the MARS system complemented with MRI and PET-CT showed tumour masses detectable in the whole abdominal cavity as multiple nodules,^17^ thus reproducing the diffusion pattern of the clinical disease, as illustrated by the representative figures in mice after 3 weeks and at the time of sacrifice (Fig. 4a). The histopathological analysis demonstrated the presence of several neoplastic lesions, and IHC revealed a strong staining for LDH-A (Fig. 4b). In keeping with these findings, photoacoustic live imaging for deep-tissue pO_2_ measurement showed reduced oxygenation, suggesting the occurrence of hypoxia in many tumour nodules (Fig. 4c).

The mice were stratified into two groups, with comparable BLI signal, and then NHI-Glc-2 was administered i.p. at the maximum tolerated dose (MTD).^40^ However, the analysis of the BLI was performed only in three animals, and the results reported in Supplementary Fig. S8, showing a 70% reduction of tumour volumes (at day 15) in animals treated with NHI-Glc-2 compared with untreated animals, should be considered with caution. The histopathological studies performed on several samples from these models (including both peritoneal tumour plaques and tumour masses in the liver) showed a clear reduction in tumour volume in treated vs. untreated animals, as well as necrotic lesions. However, F-luc requires oxygen. Accordingly, bioluminescence imaging typically underestimates hypoxic tumours, and the experiments were then repeated in groups of six animals after subcutaneous injection of 5 × 10^6^ MesoII cells. In these models, IHC showed LDH-A levels that were comparable with those observed in the orthotopic tumours (Fig. 4d). The administration of NHI-Glc-2 resulted in a significant reduction in tumour growth (Fig. 4e). At the end of the 7th week of study, animals treated with 50 mg/kg NHI-Glc-2 showed a 49% reduction in tumour mass as compared with control animals (314 vs. 620 mm^3^, *P* < 0.001). A similar decrease in tumour growth was detected in animals given gemcitabine, with a 47% reduction in tumour volume (331 vs. 620 mm^3^, *P* < 0.001). Importantly, NHI-Glc-2 was well tolerated; no toxic deaths or signs of toxicity were observed, and the body weight of animals given NHI-Glc-2 was similar to that of the drug-free controls until the end of the 7th week (Supplementary Fig. S9), whereas that of mice given gemcitabine was slightly reduced (25 vs. 28 g, *P* = 0.06). Similarly, the combination of NHI-Glc-2 and gemcitabine was not toxic and produced a significantly stronger shrinkage of the tumour mass. Indeed, statistical analyses revealed a mean reduction of fourfold in tumour volume in the animals treated with the drug combination compared with controls (123 vs. 620 mm^3^, *P* < 0.001), as well as a significant reduction compared both to animals treated with gemcitabine alone and to animals treated with NHI-Glc-2 monotherapy (*P* < 0.001).

### Correlation of LDH-A overexpression with shorter survival of MPM and DMPM patients

**Fig. 5:**
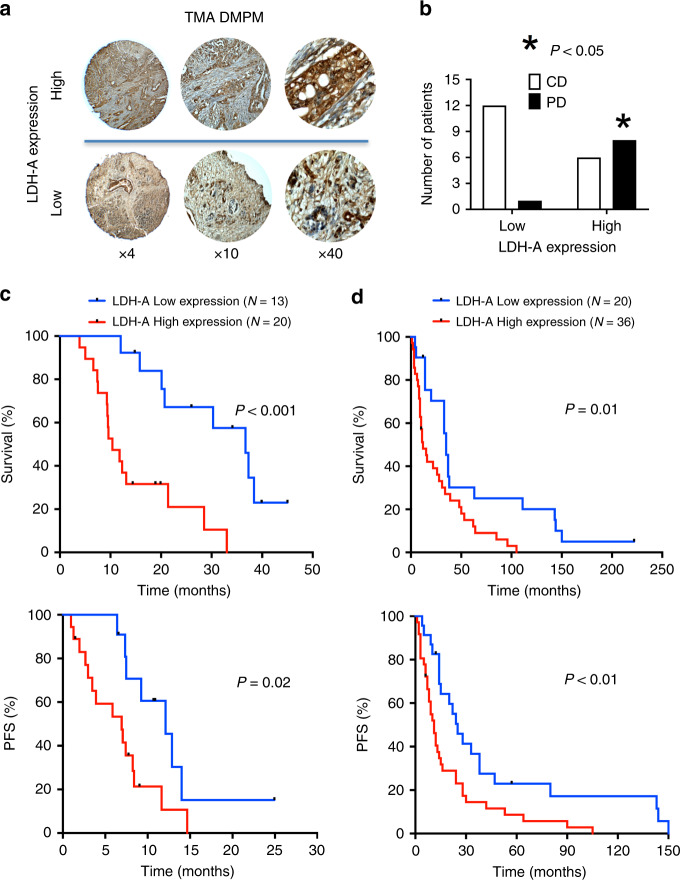
High expression of LDH-A correlates with significantly shorter overall survival (OS) and progression-free survival (PFS) in both MPM and DMPM patients. **a** Representative pictures of immunohistochemical analyses of tissue microarray (TMA) cores in the cohorts of DMPM patients, illustrating cases with high (upper panels) and low (lower panels) LDH-A expression (at ×4, ×10 and ×40 original magnification). **b** LDH expression levels correlated to Disease Control (DC) and Progressive Disease (PD) in MPM patients treated with pemetrexed-based chemotherapy. **c** Kaplan–Meier curves of OS (upper panel) and PFS (lower panel) in MPM patients according to LDH-A high vs. low expression levels, as described above. *P* values were determined with the Log-rank test. **d** Kaplan–Meier curves of OS (upper panel) and PFS (lower panel) in DMPM patients according to LDH-A high vs. low expression levels, as described above. *P* values were determined with the Log-rank test.

LDH-A protein levels were analysed by IHC on TMAs, including tumour specimens from MPM and DMPM patients (Supplementary Fig. S10; Fig. 5a). IHC staining was assessed by two independent pathologists under blinded conditions, and any discrepancies were resolved by consensus. The concordance between scores from different paraffin cores of the same tumour was greater than 90%.

These analyses showed a variable LDH-A protein expression among specimens, ranging from a few scattered positive cells with a weak cytoplasmic staining to tissues with diffuse and strong cytoplasmic reactivity, in some cases accompanied by nuclear expression. Previous studies demonstrated the hypoxic microenvironment of mesothelioma,^21,22^ and that LDH-A overexpression correlated with hypoxia in different solid tumours.^41^ In this study, 20 out of 33 MPM cases (60%) and 36 out of the 56 DMPM (64%) displayed high LDH-A expression.

Though the statistical analyses were performed on small cohorts of patients, we found a significant correlation between low LDH-A protein expression and DC after pemetrexed-based chemotherapy in MPM patients. In particular, DC was achieved in 92% of the patients with low LDH-A expression, while only 43% of patients with high LDH-A experienced DC (*P* = 0.03, Fig. 5b). The univariate analysis revealed a significant correlation between high expression levels of LDH-A and significantly shorter OS (*P* < 0.001, Fig. 5b, upper panel), and PFS (*P* = 0.02, Fig. 5c, lower panel) in MPM patients. Patients with high LDH-A expression had a median OS of 10.4 months (95% CI, 7.1–13.7) and a median PFS of 6.9 months (95% CI, 2.7–11.2), whereas patients with low expression levels of LDH-A had a median OS of 36.7 months (95% CI, 27.4–46.6) and a median PFS of 12.1 months (95% CI, 7.9–16.3). LDH-A expression levels were not associated with age, sex, histology or EORTC/PS grade. Among these clinicopathological parameters, the non-epithelioid (i.e., sarcomatoid or biphasic) histology correlated with significantly shorter OS and PFS (Supplementary Table 4), as reported previously.^1^ However, only three patients had a non-epithelioid histology, and we did not perform a multivariate analysis because of the very limited sample size.

The prognostic role of LDH-A was validated by IHC in an independent cohort of DMPM patients (Fig. 5d, upper panel). Patients with low LDH-A expression had OS of 35.0 months (95% CI, 30.7–39.3), whereas the remaining patients had an OS of 12.0 months (95% CI, 5.3–18.7, *P* = 0.01). A trend towards a significant correlation was reported for LDH-A and gender, but the latter was not correlated to survival (Supplementary Table 5). Conversely, LDH-A expression levels were not correlated with other clinicopathological parameters, including performance status (PS) and histology, which showed a correlation with both OS and PFS (Supplementary Table 5), as reported previously.^1^

A similar correlation was found for PFS. In particular, patients with low LDH-A expression had significantly longer PFS (22.0 months, 95% CI, 7.0–36.9), whereas patients with high LDH-A levels had PFS of 10.0 months (95% CI, 6.5–13.5, *P* < 0.01, Fig. 5d, lower panel). Moreover, both epithelioid subtype and good PS were associated with significantly longer PFS (Supplementary Table 5).

The Cox proportional hazard regression model used for multivariate analysis in DMPM patients confirmed LDH-A expression as an independent prognostic factor for progression and survival (Table 1). In particular, high expression levels of LDH-A were associated with an increased risk of relapse (HR = 2.4, 95% CI, 1.2–2.6, *P* = 0.01) and death (HR = 2.7, 95% CI, 1.7–3.6, *P* = 0.02).

**Table 1:** Factors associated with overall survival (OS) and progression-free survival (PFS) in the multivariate analysis of DMPM patients.

Covariates for overall survival (OS)	HR (95% CI)	Wald P
LDH: high vs. low	2.74 (1.72–3.66)	*0.02*
PS: 1–2 vs. 0	3.21 (1.91–3.82)	*0.02*
Histology: non-epithelioid vs. epithelioid	2.04 (1.13–3.14)	0.07
**Covariates for progression-free survival (PFS)**	**HR (95% CI)**	**Wald P**
LDH: high vs. low	2.41 (1.23–2.62)	*0.01*
PS: 1–2 vs. 0	3.63 (1.73–4.11)	*0.02*
Histology: non-epithelioid vs. epithelioid	2.27 (1.12–3.74)	0.29

Italic values reported the Wald P (significance was set at 0.05).

*HR* hazard ratio, *PS* performance status.

Collectively, these findings demonstrate that both MPM and DMPM are characterised by elevated expression of LDH-A, which is associated with dismal prognosis. Therefore, targeting LDH-A in these patients could constitute an attractive therapeutic avenue.

## Discussion

The current study revealed for the first time that low levels of PCFT in MPM specimens were associated with hypoxia as detected by high expression of the hypoxic marker CAIX. Moreover, we established that hypoxic and PCFT-silenced cells are characterised by upregulation of LDH-A.

We then explored the clinical relevance of this discovery, and reported the key prognostic value of LDH-A in both MPM and DMPM patients. Furthermore, we used innovative in vitro and in vivo models to characterise the anti-proliferative capacity of LDH-A inhibitors, in order to provide mechanistic insights regarding the aggressive behaviour of mesothelioma, and in an attempt to contribute to the rational development of new prognostic and therapeutic approaches.

PCFT has recently emerged as a predictive parameter in MPM patients treated with pemetrexed-based chemotherapeutic regimens, since low PCFT expression was associated with shorter PFS and OS.^13^ PCFT is the main transporter mediating pemetrexed influx, with remarkable transport Km values of 0.2–0.8 μM at acidic pH,^42^ and its expression has been linked to growth-inhibitory activity of pemetrexed after transfection into a folate transporter-null cell variant.^43^ We consistently observed that PCFT silencing increased the IC_50_ values of pemetrexed in our MPM cell lines.

We have previously shown the regulation of PCFT gene expression by NRF-1,^38^ the dominant transcription factor orchestrating mitochondrial biogenesis and respiration, which acts as a key repressor of HIF-1α under hypoxic conditions.^44^ Similarly, we previously found that hypoxia downregulated gene expression of both RFC and PCFT, as well as other key enzymes in folate metabolism, and rendered HeLa cells refractory to both pemetrexed and other hydrophilic and lipophilic antifolates.^14^ However, the present study is the first to demonstrate the role of PCFT suppression and hypoxia as causative of pemetrexed resistance in MPM. Indeed, we observed in MPM specimens that low/intermediate levels of expression of the hypoxic marker CAIX were associated with intermediate expression levels of PCFT, while specimens with the highest levels of CAIX showed reduced PCFT expression. In line with these results, in vitro studies under hypoxic conditions and in mesothelioma spheroids showed that the levels of PCFT expression were reduced, accompanied by decreased pemetrexed activity. Using these in vitro models of hypoxia, along with PCFT-silenced cells, and a specific PCR array, we found that LDH-A was the only significantly upregulated gene in all three models.

The oxidoreductase LDH-A is a key enzyme in aerobic glycolysis, as well as a major checkpoint for the switch from aerobic to anaerobic glycolysis, which catalyses the reduction of pyruvate to lactate, and constitutes a fundamental metabolic adaptation of cancer cells to a relatively hostile environment, facilitating tumour growth.^32,45^ Serum, plasma and tissue levels of LDH-A are prognostic factors in several tumour types, and the ratio of pleural fluid to serum LDH > 1.0 was a significant predictor for OS in 71 MPM patients.^46^ Moreover, gene expression analysis of 16 MPM specimens compared with four control pleural tissue samples using cDNA microarray filters with 4132 clones showed that LDH-A was among the 166 significantly upregulated genes.^47^ In particular, the expression of LDH-A showed a 5.5-fold change (*P* = 0.00001), which was validated by RT-PCR. Interestingly, the analysis of our microarray data showed a significantly higher expression of LDHAL6A in DMPM tissues compared with normal mesothelial tissues, and this protein has all the biological functions of LDH-A according to KEGG and REACTOME databases.

Lactate production also contributes to extracellular acidosis, thus supporting tumour invasiveness and exerting immunosuppressive effects.^45^ This has raised interest regarding the mechanisms underlying regulation of LDH-A in cancer cells, including critical post-transcriptional modifications.^48,49^ Importantly, the results of the present study also suggest that low PCFT expression levels might directly or indirectly stimulate the enhanced expression of LDH-A, though the mechanism by which this occurs remains unclear. Increased acidity caused by LDH-A could in principle favour folate transport via PCFT, even when the latter is expressed at decreased levels, and this should enhance pemetrexed uptake.

Notably, in this study, LDH-A emerges also as an attractive druggable target for therapeutic interventions. Previous studies with the LDH inhibitor oxamate on glioma spheroids have shown the changed metabolism with drastic changes in radiation sensitivity.^50^ In the present study, the specific LDH-A inhibitor NHI-2 was cytotoxic to H2452 MPM spheroids under conditions where pemetrexed was ineffective. Similarly, a glycoconjugated analogue of NHI-2 (NHI-Glc-2) was active against MPM and DMPM primary cells, growing both as monolayers and as spheroids. Because of its enhanced uptake into tumour cells, likely mediated by glucose transporters (GLUTs),^27^ NHI-Glc-2 was also selected for in vivo studies. Several studies suggested that early passages of primary tumour cells and “avatar” mice can mimic the genetic diversity that characterises the human disease, and represent the best preclinical platform to study drug activity in different tumour types, including mesothelioma.^51,52^ However, there is still a need for experimental models coupled to in-depth molecular profiling in order to decipher the genetic alterations that drive drug sensitivity/resistance, and only a few studies evaluated DMPM models. Therefore, in a proof-of-principle pharmacological study, we used our recently established mouse models, obtained by orthotopic or subcutaneous inoculation of primary DMPM cells, genetically engineered to express the F-luc luciferase, providing an ease-of-use, low-cost, non-invasive and high-throughput imaging tool to monitor tumour growth.^24^ These models recapitulated the main histological and genetic features of the primary tumours, including hypoxic tumour domains and overexpression of LDH-A;^21,47^ remarkably, NHI-Glc-2 caused a significant reduction in tumour growth, when compared with untreated animals. Moreover, the in vivo studies on the combination of NHI-Glc-2 with gemcitabine reflected the synergistic effects observed in vitro, revealing a significant reduction compared with animals treated with either gemcitabine or NHI-Glc-2. These results are consistent with our previous findings on the synergistic interaction of gemcitabine with other LDH-A inhibitors in preclinical models of pancreatic cancer and MPM.^17,18^ Further studies are warranted to unravel the molecular mechanisms underlying this synergistic interaction. However, one may speculate that hypoxia can affect gemcitabine activity, as was found in other 3D models.^53^ The synthesis of active gemcitabine deoxynucleotides was possibly decreased through downregulation, genomic deletions and frame-shift mutations of the rate-limiting enzyme deoxycytidine kinase, as reported recently for acute myeloid leukaemia and pancreatic cancer cells,^17,54,55^ or downregulation of the equilibrative nucleoside transporter 1, as reported previously in MPM cells.^18^ However, since hypoxic cells represent only a subpopulation in most tumours, and anti-hypoxia agents require a long residence time in tumours in order to exploit fluctuating hypoxia and cause efficient, long-range, bystander killing,^56^ this synergistic activity is extremely important. Moreover, in agreement with the hypothesis that inhibition of molecular targets in hypoxic cells should offer a more benign toxicity profile, which is distinctly different from that of conventional cytotoxic therapy, we indeed did not observe untoward toxicity in animals treated with a combination of gemcitabine and NHI-Glc-2. These results prompt further studies on the combination of our new anti-LDH-A agents with current standard cytotoxic agents in clinical trials.

Another important step we undertook towards the evaluation of the role of LDH-A in the clinical setting was the analysis of the correlation of its expression with clinical outcome. A recent meta-analysis showed that elevated serum LDH-A levels prior to chemotherapeutic treatment, are a significant prognostic factor in malignant mesothelioma.^57^ However, to the best of our knowledge, the current research is the first study to demonstrate the prognostic role of LDH-A protein expression in tumour tissues from both MPM and DMPM patients.

Our current study is inarguably not a long-term follow-up and prolonged longitudinal study of the impact of treatment. On the other hand, in other animal models, it has already been shown that tumour growth in the first 7–10 days post engraftment, can reliably predict the effect of treatment and overall survival.^58^ In addition, this non-long-term follow-up in our models would alleviate significant genetic alterations as compared with the original tumours.

An important limitation of our study is that LDH-A expression was tested retrospectively in relatively small cohorts of MPM and DMPM patients, treated with different therapies with and without pemetrexed, and on a small number of mesothelioma cell lines and primary cells. However, bench-to-bedside research on hundreds of samples, which clearly improved prognostic capabilities in several tumour types, such as lung or breast cancer, is extremely difficult to achieve in mesotheliomas, which are rare tumours.^1^ Indeed, most previous candidate biomarkers are based solely on mRNA evidence, and our findings with protein levels in two cohorts of patients, indicate the value of the prognostic value of LDH-A, and should prompt prospective studies for further validation. Moreover, our data showed that high expression levels of LDH-A are detectable in more than 50% of specimens from both MPM and DMPM patients. Thus, these findings might be relevant to a large number of mesothelioma patients.

A large number of compounds with LDH-A inhibitory activity have been discovered and studied pre-clinically as anticancer agents,^45^ none of which is currently approved for clinical use. In addition, many trials with new, targeted agents resulted in clinical failures in mesothelioma patients.^59,60^ However, a combined approach of chemical biology, preclinical pharmacology, bioinformatics and appropriate models and biobanks, as reported in this study, should create new opportunities for the development of more effective inhibitors as drug-like candidates.

In conclusion, our clinical data, combined with our in vitro and in vivo findings, strongly suggest that mesothelioma is more aggressive if it has high expression of LDH-A, which can be targeted by specific LDH-A inhibitors, therefore representing a promising new avenue for prognostic and therapeutic purposes.

## Supplementary information

Supplemental methods & results

## Data Availability

All data generated or analysed during this study are included in this published article (and in supplementary information files).
