# Supplementary material for: Impact of hypoxia on chemoresistance of mesothelioma mediated by the proton-coupled folate transporter, and preclinical activity of new anti-LDH-A compounds
Source: Br J Cancer. 2020 Jun 4;123(4):644–56. doi: 10.1038/s41416-020-0912-9 (PMC7434895; doi:10.1038/s41416-020-0912-9)
Supplement: Supplementary file 1 — Supplemental methods & results [file 41416_2020_912_MOESM1_ESM.pdf]

Supplemental methods

Patients, TMAs and Immunohistochemistry (IHC). Patient selection for the tissue-microarrays (TMAs) was based on the following inclusion criteria: a histological diagnosis of MPM or DMPM, and the availability of tumor tissue and clinicopathological data, which were recorded in prospectively maintained databases and anonymized after inclusion.

The study protocols were approved by the institutional review boards and the ethics committees of the Humanitas Research Hospital, Rozzano, Milan, Italy and National Cancer Institute, Milan, Italy, in accordance with the ethical guidelines of the Declaration of Helsinki. All participants provided written informed consent for sample collection and subsequent analysis.

For the TMA with MPM samples, the paraffin-embedded surgical or biopsy specimens (slides of 10 µm) were collected from the pathology files of the Humanitas Research Hospital, Rozzano, Milan, Italy (from January 2008 to December 2013). All these samples were collected before chemotherapy. All patients received pemetrexed at 500 mg/m^2 and a carboplatin infusion with a target area under the plasma concentration–time curve of 5 mg/mL*minutes (AUC5), administered intravenously every 21 days. All patients received folic acid and vitamin B12 supplementation. Patient characteristics were described in terms of number and percentage, or median and range, when appropriate, according to a recent prognostic study on a cohort of unresectable MPM patients who received pemetrexed-based chemotherapy [Bille' et al., 2017].

For the TMA with DMPM samples, tumor tissues of 56 DMPM patients treated with

CRS and HIPEC in the National Cancer Institute, Milan, Italy (from August 1995 to October 2013) were selected for pathological examination. TMAs were constructed by using a tissue-arraying instrument (Beecher Instruments, Silver Springs, MD, USA). IHC staining of paraffin-embedded tissues from MPM patients for proton-coupled folate transporter (PCFT), carbonic anhydrase IX (CAIX) and lactate dehydrogenase (LDH-A) were performed as described previously. Before staining with specific polyclonal rabbit anti-human antibody for PCFT [Hou et al, 2012], or with the commercial anti-CAIX (dilution 1:500; monoclonal antibody ab15086, Abcam, Cambridge, MA) and anti-LDH-A LDH-A (dilution 1:100; ab9002, Abcam) antibodies, the TMA slides were deparaffinized using xylene, rehydrated in alcohol and microwaved at 400W (2 times, for 5 minutes). Immunostaining was performed by the avidin-biotin peroxidase complex technique. Negative controls were obtained by replacement of primary antibody with phosphate-buffered saline (PBS), while positive controls were obtained using sections of colorectal cancer. Immunoreactivity was enhanced by antigen retrieval for 30 minutes in 10 mM sodium citrate, pH 6.0. The sections were washed three times in PBS and blocked with Super Block (Skytek Laboratories, Logan, UT) and 3% hydrogen peroxide for 10 minutes prior to overnight incubation at 4°C with the primary antibody (dilution, 1:40). After overnight incubation with the primary antibodies, the sections were washed two times for 3 minutes in PBS and incubated with the appropriate kit containing the secondary antibody tagged with avidin-biotinylated horseradish peroxidase (Cell Marque revelation Kit, Sigma). Finally, the colorimetric reaction obtained with 3'-3' diaminobenzidine was counterstained with hematoxylin, and slides were permanently fixed with synthetic mounting. The sections were scored by two researchers blinded

to clinical outcome, who also evaluated the extent of tissue loss, background staining and overall interpretability. Scoring for PCFT was described in our previous study [Giovannetti et al., 2017]

Immunostaining intensity of CAIX was described in the supplemental table 1, while for LDH-A we used a previously proposed grading system with two LDH-A expression levels: strong cytoplasmic expression in >50% of cancer cells or nuclear expression in >10% of cancer cells was defined as high expression; otherwise, nuclear expression was considered low [Koukourakis et al., 2006].

Neoplastic cells were always uniformly stained and counting all the tumor cells in each spot made positivity assessment. To further implement the reproducibly of our technical procedures, as discussed previously for other biomarkers in MPM samples [Li Petri et al., 2018], all stained TMA sections were also digitally imaged at 40X, using a computerized high-resolution acquisition system (D-Sight, Menarini, Florence, Italy), equipped with the automated quantitative image analysis software algorithm DSight software 2.1.0. Multipreview of the images allowed editing of the area of interests.

Determination of Mitochondrial Function and Glycolysis in Mesothelioma cells

Oxygen Consumption Rate (OCR) and ECAR (Extracellular Acidification Rate) were measured in the mesothelioma cell lines MSTO and H2452 by using the Seahorse XFp Metabolic Flux Analyzer (Agilent Technologies, Inc, Santa Clara, Ca, USA), as described previously [Schipper et al, 2017]. One day before the assay, cells were seeded at a density of 40,000 per well in a final volume of 80 µl in a Seahorse plate. The mitochondrial stress test was performed according to manufacturer's instructions. Before the analysis, medium was changed with Seahorse XFp RPMI medium, enriched

with 1 mM pyruvate, 2 mM glutamine and 10 mM glucose and then incubated for 45 minutes. After three baseline measurements, Oligomycin (inhibitor of V Complex), Carbonyl cyanide-4 (trifluoromethoxy) phenylhydrazone (FCCP, mitochondrial uncoupler) and a mix of Rotenon and Antimycin A (inhibitors of Complexes I and III) were sequentially injected into each well to final concentrations of 1.5 µM, 0.5 µM 0.5 µM. The obtained data allowed for calculations of ATP-linked Respiration, Maximal Respiration, Spare Capacity, Proton Leak and Non-Mitochondrial Oxygen Consumption. The Glycolysis Stress Test was conducted with similar seeding conditions. At the day of analysis, the Seahorse XFp RPMI medium was enriched with 2 mM glutamine and adjusted to pH 7.4. Glucose (substrate for glycolysis), Oligomycin (inhibitor of mitochondrial ATP production) and 2-deoxyglucose (inhibitor of hexokinase) were consecutively added into wells to final concentrations of 10 mM, 5 µM and 100 mM, respectively. The Glycolytic capacity, Glycolytic reserve, Glycolysis and Non-Glycolytic Acidification were estimated and normalized to mg of protein.

Quantitative-RT-PCR (qRT-PCR). PCR reactions were performed in triplicate with 5 µL of cDNA, 12.5 µL of TaqMan Universal PCR Master Mix, and 5 µL of probe, and forward and reverse primers in a final volume of 25 µL. Samples were amplified by the following thermal profile: an initial incubation at 50°C for 5 minutes to prevent the reamplification of carry-over PCR products by AmpErase uracil-N-glycosylase, followed by incubation at 95°C for 10 minutes to supress AmpErase UNG activity and denature the DNA, 50 cycles of denaturation at 95°C for 15 seconds, followed by annealing and extension at 60°C for 1 minute. Specific forward and reverse primers and probes were obtained from Applied Biosystems Assay-on-Demand products. All

the samples were amplified in triplicate with appropriate non-template controls, and the coefficient of variation was less than 2%.

Analysis of *PCFT* gene expression modulation by siRNA. For the analysis of PCFT modulation, MPM cells were plated in triplicate at a density of 2×10^5 cells/well in 6-well plates. After 24 hours, the cells were treated with siRNA anti-PCFT or negative control (Silencer® Select Negative Control #1 siRNA, Ambion) in a final RNA concentration of 25 nmol/L. Lipofectamine™ was used as transfection solution, according to the manufacturer's instructions. The modulation of *PCFT* expression by this siRNA was investigated by q-RT-PCR, after 48 and 72 hours, as described previously [Maftouh et al., 2015].

Cell growth inhibition assays. For sulforhodamine-B (SRB) assays, cells were plated at $5x10^4$ cells per well, using 96-well plates, and growth inhibition was expressed as the percentage of control (vehicle treated cells) absorbance (corrected for absorbance before drug addition). After 72 hours, optical density was measured at 540 nm using the Tecan SpectraFluor (Tecan, San Diego, CA, USA). The 50% inhibitory concentration of cell growth (IC50) was calculated by non-linear least squares curve fitting (GraphPad PRISM version 5.0, Intuitive Software for Science, San Diego, CA, USA). To investigate whether the modulation of PCFT expression affected pemetrexed cytotoxicity, we performed the same SRB experiments in cells treated for 48 hours with the siRNA anti-PCFT or its negative control, in a final RNA concentration of 25 nmol/L.

Spheroids. MPM spheroids were established by seeding 1000 MPM cells per ml in

DMEM/F12 GlutaMAX-I (1:1, Invitrogen), in ultra-low attachment plates (Corning Incorporated, Corning, NY). These spheroids were generated for 7-10 days, and then harvested for RNA isolation and analysis of PCFT expression, as described above. Spheroid volume (V) was calculated from the geometric mean of the perpendicular diameters D = (Dmax + Dmin)/2, as follows: V = (4/3) * π(D/2)3).

We also performed an exploratory analysis using a sequential trypsin digestion of spheroids of H2452 that had reached a diameter of approximately 500 µm. These serial trypsin treatments, enabled the segregation into four heterogeneous populations comprising proliferating cells from the surface (SL), an intermediate region (IR), nonproliferating hypoxic cells from the predominantly hypoxic perinecrotic region (PN), and a necrotic core (NC), as described previously [McMahon et al., 2012]. Aliquots containing cells in suspension derived from pooled NC/PN versus SL/IR regions were segregated and 10^5 viable (trypan blue excluding) cells were plated into 6-well culture dishes and treated with PMX 1 and 10 µM. After 7 days incubation at 37 °C, the drug-treated colonies of greater than 50 cells were counted and plating efficiency determined as the number of colonies formed/number of cells plated expressed as a percentage of untreated colonies.

In parallel experiments we also evaluated whether pemetrexed was able to affect spheroid formation by counting the number of spheroids created in cells exposed immediately after seeding to 0.1, 1, 10 and 20 µM pemetrexed for 72 hours, compared to untreated cells.

MESO II and STO spheroids with a diameter of approximately 300 µm were created in 96-well flat bottom plates coated with 1.5% agarose. DMEM/F12 medium was replaced with drug-free medium or medium containing gemcitabine or PI-FLY161 (6

wells per condition). Images of spheroids were taken with an automated phase-contrast microscope (Universal Grab software, Digital Cell Imaging Labs). To detect the amount of light passing through the spheroids, pixel intensities of 8-bit black/white-converted images were calculated using ImageJ Software (U.S. National Institutes of Health, Bethesda, Maryland, USA) and expressed as Mean Grey value (= sum of all Grey values of the spheroid selection divided by the pixels of that selection). Inhibition of cell aggregation for each drug-treated spheroid after 7 days ("treated" in the formula below) was calculated by normalizing for the Mean Grey values of the sum control spheroids (Σ control, where "n" is the number of replicates in the formula below) as follows: Inhibition of cell aggregation = $|((1 - treated)/(\Sigma control$ / n))|

Western blot of LDH-A. In order to evaluate the modulation of LDH-A protein expression in MPM cells growing as a monolayers or as spheroids, the H28, H2452 and MSTO-211H cells were cultured for 72 h and western blotting was performed, as described earlier [Maftouh et al, 2012]. Briefly, 30 30 µg of proteins was separated on a 10% SDS- polyacrylamide gel and transferred onto PVDF membrane (Immobilion-FL, Millipore, Billerica, MA). The membrane was blocked with Rockland (Rockland Inc., Boyertown, PA), and incubated overnight with anti-LDH-A antibody (Abcam, Cambridge, UK, at 1:1000 dilution), and mouse anti-β-actin (1:10000 dilution; Sigma).

***In vivo* experiments using orthotopic and subcutaneous mouse models and live imaging**

In vivo experiments were performed in nu/nu athymic female mice 4 weeks old with average 23 g of weight (range, 22-24 g) at the arrival, while the weight during the

experiment is reported in the Supplemental Figure S8). The animals were purchased purchased from Harlan (Horst, The Netherlands).

The working protocol was approved by the local ethical committees on animal experimentation of the VU University Medical Center (VUmc, Amsterdam, The Netherlands) and of the University of Pisa (Comitato di Ateneo per la Sperimentazione Animale, Pisa, Italy), according to the 2010/63/EU European Community Council Directive for laboratory animal care.

In the study design and report we followed the ARRIVE reporting guideline and provide a completed ARRIVE checklist as a supplemental file specifying where in the manuscript each item is reported.

In particular, we undertook the following steps to minimise the effects of subjective bias when allocating animals to treatment: randomisation procedure (matching for tumor volume and animal weight) and blinding assessment of the results by the pathologists. The animals were hosted in cages (3 animals/cage), under pathogen free [SPF] condition, with standard light/dark cycle, and temperature conditions, and free access to food and water, environmental enrichment). Welfare-related assessments and interventions were carried out prior to, and during the experiment.

Orthotopic primary DMPM models (n=5 tumors per treatment group) were generated by injection of 3×10^6 Fm/GC primary cells into the peritoneal cavity of the mice. Postoperative pain was counteracted by administering temgesic (0.05-0.1 mg/kg SC), which was already demonstrated to be an effective anaesthetic [Giovannetti et al., 2014]. Mice were treated with NHI-Glc-2, solubilized in Polyethylene glycol 400 (PEG400, Sigma-Aldrich, St. Louis, MO), at 100 mg/kg, 5 days (1-5) for 2 weeks (formulation concentration: 25 mg/mL in PEG400, 100 µL i.p. injection for a 25 g

mouse). The drug was administered in the morning, in the mouse facility laboratory. the animals in the different experimental groups were treated and assessed always in the same order. Lidocaine was used as local anesthetic on the skin of the mouse.

Bioluminescence imaging (BLI) was evaluated with a Bruker In-Vivo Xtreme Capture System, using Molecular Imaging Software (Bruker Corporation, Billerica, MA).

Additional imaging analyses to define tumor spatial characteristics and evaluate microenvironment structures, such as neovasculature and hypoxic status, were carried out by high-frequency-ultrasound including Power Doppler Mode (Vevo-2100, VisualSonics, Amsterdam, The Netherlands).

Further experiments were performed on subcutaneous tumors, obtained by inoculation of 3×10^6 tumor cells. When tumor volume reached an average size of 100 mm^3, the animals were randomly distributed into 4 groups (n=6 tumors per treatment group) as follows: 1) control/untreated mice; 2) mice treated with gemcitabine alone at 100 mg/kg, 2 days (day 1 and 4) for three weeks (formulation concentration: 25 mg/mL in PBS, 100 μL i.p. injection for a 25 g mouse); 3) mice treated with NHI-Glc-2, solubilized in PEG400, at 50 mg/kg, 5 days (day 1-5) for three weeks (formulation concentration: 12.5 mg/mL in PEG400); and 4) mice treated with a simultaneous combination of gemcitabine and NHI-Glc-2, at the doses mentioned above, for three weeks. Tumor xenografts were measured as described previously [Cavazzoni et al., 2017]. No deaths and 100% tumor take-rate are reported in the literature for mesothelioma models obtained by IP injection of mesothelioma cells. Research papers report mouse death essentially due to tumor and metastasis development. However, our mice were sacrificed before MM primary tumors and metastases can cause severe symptoms, via cervical dislocation. Since previous studies showed that

orthotopic administration of cancer cell lines gives tumor growth in 100% [Pinton et al., 2014] we used a number of 3 mice per group (+two extra mouse in case of sudden death) to test if a primary cell culture results in tumor formation. The number of animals for the analysis of drug activity in the subcutaneous models was based on previous studies in which a standard error of 34% was reported in the number of proliferating cancer cells [Shah et al, 2011]. With this standard error, a group size of 6 animals is sufficient to detect a treatment effect of 55%.

Regarding the implications of our experimental methods or findings for the replacement, refinement or reduction (the "3Rs") of the use of animals in research we declared in our research protocol that "The development of a mouse model with mesothelioma (MM) primary cultures is of crucial importance to gain a better understanding of the biology of this devastating tumor. Furthermore, such models will allow preclinical research into new therapeutic strategies. The MM animal models will be used to test the effects of drugs potentially effective in treating patients affected by this devastating disease. So far, many trials have been initiated in the clinic without supporting evidence from pre-clinical research or using traditional preclinical models that deviate from their originator tumor. The lack of such pre-clinical studies with appropriate mice models may be one of the causes of the lack of positive results in clinical trials, and ultimately for the lack of improvement in survival expectancy in these patients. The use of animals and the moderate/severe distress that will be caused to them is acceptable in the light of the extreme need for research into the biology of MM. Furthermore, we strongly believe that our experiments will allow us to develop novel effective therapeutic strategies for patients affected by this aggressive and lethal tumor." Moreover, our scientific findings, based on

appropriately designed and analysed animal experiments, should further accelerate the development and use of models and tools, based on the latest science and technologies, to address important scientific questions and have impact of welfare minimising the use of animals in future studies.

Supplementary References (in alphabetic order)

- Billé A, Krug LM, Woo KM, Rusch VW, Zauderer MG. Contemporary Analysis of Prognostic Factors in Patients with Unresectable Malignant Pleural Mesothelioma. J Thorac Oncol. 2016;11:249–55.
- Enhanced efficacy of AKT and FAK kinase combined inhibition in squamous cell lung carcinomas with stable reduction in PTEN.
- Cavazzoni A, La Monica S, Alfieri R, Ravelli A, Van Der Steen N, Sciarrillo R, et al. Oncotarget. 2017;8(32):53068-53083.
- Giovannetti E, Wang Q, Avan A, Funel N, Lagerweij T, Lee JH, et al. Role of CYB5A in pancreatic cancer prognosis and autophagy modulation. J Natl Cancer Inst. 2014;106(1):djt346.
- Giovannetti E, Zucali PA, Assaraf YG, Funel N, Gemelli M, Stark M, et al. Role of proton-coupled folate transporter in pemetrexed resistance of mesothelioma: clinical evidence and new pharmacological tools. Ann Oncol 2017;28:2725–32.
- Hou Z, Kugel Desmoulin S, Etnyre E, Olive M, Hsiung B, Cherian C, Wloszczynski PA, Moin K, Matherly LH. Identification and functional impact of homo-oligomers of the human proton-coupled folate transporter. J Biol Chem. 2012;287:4982-95.
- Koukourakis MI, Giatromanolaki A, Sivridis E, Gatter KC, Harris AL; Tumour Angiogenesis Research Group. Lactate dehydrogenase 5 expression in operable

colorectal cancer: strong association with survival and activated vascular endothelial growth factor pathway--a report of the Tumour Angiogenesis Research Group. J Clin Oncol. 2006;24(26):4301-8.

- Li Petri, Cascioferro S, Parrino B, Peters GJ, Diana P, Giovannetti E. Proton-coupled folate transporter as a biomarker of outcome to treatment for pleural mesothelioma. Pharmacogenomics. 2018;19(10):811-814.
- Maftouh M, Avan A, Sciarrillo R, Granchi C, Leon LG, Rani R, et al. Synergistic interaction of novel lactate dehydrogenase inhibitors with gemcitabine against pancreatic cancer cells in hypoxia. Br J Cancer. 2014;110(1):172-82.
- McMahon KM, Volpato M, Chi HY, Musiwaro P, Poterlowicz K, Peng Y, et al. Characterization of changes in the proteome in different regions of 3D multicell tumor spheroids. J Proteome Res. 2012;11(5):2863-75.
- Pinton G, Manente AG, Daga A, Cilli M, Rinaldi M, Nilsson S, Moro L. Agonist activation of estrogen receptor beta (ERŒ≤) sensitizes malignant pleural mesothelioma cells to cisplatin cytotoxicity. Mol Cancer. 2014;13:227.
- Schipper DA, Palsma R, Marsh KM, O'Hare C, Dicken DS, Lick S, Kazui T, Johnson K, Smolenski RT, Duncker DJ, Khalpey Z. Chronic Myocardial Ischemia Leads to Loss of Maximal Oxygen Consumption and Complex I Dysfunction. Ann Thorac Surg. 2017;104(4):1298-1304.
- Shah N, Zhai G, Knowles JA, Stockard CR, Grizzle WE, Fineberg N, et al. 18)F-FDG PET/CT imaging detects therapy efficacy of anti-EMMPRIN antibody and gemcitabine in orthotopic pancreatic tumor xenografts. Mol Imaging Biol. 2012;14(2):237-44.

Supplemental Table 1. Baseline characteristics of MPM and DMPM

**Clinical characteristics**	***n* of patients (%)**	
	**MPM (N=33)**	**DMPM (N=56)**
**Age**, years [median, range]	68 [47-82]	73 [51-85]
Age		
≤65	13 (39)	28 (50)
>65	20 (61)	28 (50)
**Gender**		
Male	16 (48)	17 (30)
Female	17 (52)	39 (70)
**EORTC prognostic score / PS**		
Good / 0	28 (85)	44 (78)
Poor /1-2	5 (15)	12 (22)
**Histologic subtype**		
Epithelial	30 (90)	47 (84)
Non-epithelial	3 (10)	9 (16)
PFS, months (median, 95%CI)	7.5 (6.2-8.8)	13.0 (10.2-15.7)
OS, months (median, 95%CI)	20.1 (8.5-31.7)	26.0 (11.9-40.1)

Notes: Survival and PFS data were available for all patients. Abbreviations: OS, Overall survival; PFS, progression-Free survival; PS, performance status

Supplemental table 2. Scoring of CAIX protein expression staining

Intensity of staning			Weak	Middle	Strong
	Range	Score	1	2	3
Positive cells %	X=0	0	NO CAIX	NO CAIX	NO CAIX
	0<x<25	1	Low CAIX	Low CAIX	High CAIX
	25<x<	2	Low CAIX	High CAIX	High CAIX
	x>75	3	High CAIX	High CAIX	High CAIX
"High CAIX" include the scores 4,5 and 6<br>"Low CAIX" includes the scores 2 and 3					

Supplemental table 3. List of genes significantly up-regulated in cells growing as monolayers or spheroids, after exposure to hypoxic conditions as well as after PCFT silencing (showing a PCFT reduction of 80% at RT-PCR) and, using the Hypoxia RT2 ProfilerTM array. The genes are reported in alphabetic order (NC, negative control, genes in blue are in common between the "Spheroids vs. monolayers" and the "Hypoxia vs. Normoxia" groups, genes in green are in common between the "Spheroids vs. monolayers" and the "PCFT siRNA vs. NC siRNA" groups, genes in red are in common between the "Hypoxia vs. Normoxia" and the "PCFT siRNA vs. NC siRNA" groups, **LDH-A** is the only gene in common among all these three groups).

P values <0.01; Fold change= 3		
Spheroids vs. monolayers N=25	Hypoxia vs. Normoxia N=49	PCFT siRNA vs. NC siRNA N=14
ADM	*ADORA2B*	*ATR*
ALDOA	*ANGPTL4*	*BLM*
ARNT	*ANKRD37*	*BHLHE40*
BTG1	*ANXA2*	*BNIP3*
ENO1	*APEX1*	*CA9*
HIF1A	*ARNT*	*EGLN1*
HIF3A	*ATR*	*FOS*
HK2	*BNIP3*	*HIF1A*
HNF4A	*BNIP3L*	*IGFBP3*
LDH-A	*BTG1*	***LDH-A***
LGALS3	*CA9*	*MMP9*
LOX	*CCNG2*	*MET*
MAP3K1	*COPS5*	*NFKB1*
MET	*CTSA*	*PFKP*
MIF	*DDIT4*	
MMP9	*DNAJC5*	
NCOA1	*EDN1*	
NFKB1	*EGLN2*	
PDK1	*EGR1*	
PFKFB4	*EPO*	
PGK1	*GBE1*	
PGK1	*GBE1*	
PIM1	*HIF3A*	
PKM2	*HNF4A*	
SLC2A1	*IER3*	
SLC2A3	*IGFBP3*	
	JMJD6	
	LDH-A	
	LOX	
	MIF	
	MXI1	
	NDRG1	
	NOS3	
	ODC1	
	P4HA1	
	P4HB	
	PKM2	
	PLAU	
	RBPJ	
	RUVBL2	
	SERPINE1	
	SLC2A1	
	SLC2A3	
	TFRC	
	TPI1	
	TXNIP	
	USF2	
	VDAC1	
	VEGFA	

Supplemental Table 4. Outcome of the patients in the MPM patients according to clinicopathological characteristics.

**Characteristics**	**LDH-A expression**		**PFS (Months)**	**OS (Months)**
	Low	*High*	*Median (95%CI)*	*Median (95%CI)*
			7.5 (6.2-8.8)	20.1 (8.5-31.7)
Age				
≤ 65	6	7	8.4 (5.2-11.6)	21.4 (2.2-40.6)
> 65	7	13	7.5 (6.2-8.8)	13.1 (8.4-31.7)
p-value	*0.52*		*0.91*	*0.32*
Gender				
Female	6	10	8.2 (7.0-9.4)	15.8 (4.9-26.7)
Male	7	10	7.4 (4.7-10.1)	20.1 (3.5-36.7)
p-value	*0.83*		*0.61*	*0.84*
EORTC prognostic score				
Good	*13*	*15*	7.7	20.6
Poor	*0*	*5*	6.8	19.1
p-value	*0.14*		*0.91*	*0.32*
Histology				
Epithelioid	13	17	8.4 (6.0-10.8)	20.7 (12.8-28.7)
Non-epithelioid	0	3	5.9 (0.8-11.0)	6.6 (2.3-10.9)
p-value	*0.40*		*0.02*	*0.04*

Abbreviations: EORTC, European Organization for Research and Treatment of Cancer; PFS, progression-free survival; OS, overall survival (Survival data were available for all patients and the minimum follow-up at the time of analysis was 27 months).

Notes: The potential association of LDH-A expression with clinicopathological characteristics was compared between groups using the chi-square test, with continuity correction when cells had expected count less than 5, while the correlation with PFS and OS was evaluated using the log-rank test

Supplemental Table 5. Outcome of the patients in the DMPM patients according to clinicopathological characteristics.

**Characteristics**	**LDH-A expression**		**PFS (Months)**	**OS (Months)**
	Low	*High*	*Median (95%CI)*	*Median (95%CI)*
			13.0 (10.2-15.7)	26.0 (11.9-40.1)
Age				
≤ 65	9	19	10.0 (4.8-15.2)	14.0 (11.9-36.0)
> 65	12	16	14.0 (10.1-17.9)	31.0 (15.2-46.8)
p-value	*0.41*		*0.49*	*0.57*
Gender				
Female	18	21	14.0 (12.0-16.0)	26.0 (9.6-42.4)
Male	3	14	10.0 (7.3-12.7)	22.0 (3.8-40.2)
p-value	*0.08*		*0.52*	*0.53*
PS				
0	*17*	*27*	14.0 (5.3-22.7)	31.0 (19.6-42.4)
1-2	*4*	*8*	8.0 (4.8-15.9)	8.0 (6.9-18.2)
p-value	*0.74*		*<0.01*	*<0.01*
Histology				
Epithelioid	19	25	14.0 (11.8-16.2)	31.0 (17.0-45.0)
Non-epithelioid	2	7	8.0 (4.0-16.8)	9.0 (6.1-11.9)
p-value	*0.43*		*0.07*	*0.02*

Abbreviations: PFS, progression-free survival; PS, Performance Status; OS, overall survival.

Notes: The potential association of LDH-A expression with clinicopathological characteristics was compared between groups using the chi-square test, with continuity correction when cells had expected count less than 5, while the correlation with PFS and OS was evaluated using the log-rank test

Legends to the Supplemental Figures

Figure S1: Differential profiles in the metabolism of MPM cells. The MSTO-211H and H2452 mesothelioma cells showed different profiles in their basal energy metabolism as assessed by using Seahorse XF24 Extracellular Flux analyser, as described previously [Schipper et al., Ann Thorac Surg. 2017]. Specifically, this machine measures glycolysis by analysing the extracellular acidification rate (ECAR) and measures mitochondrial oxidative phosphorylation on the basis of the oxygen consumption rate (OCR), through real-time and live cell analysis. The OCR of MSTO-211H and H2452 mesothelioma cells was measured after Seahorse XFp Cell Mito Stress Test over time (minutes), and resulting data calculated for ATP-linked basal Respiration, maximal respiration, spare capacity, non-mitochondrial oxygen consumption and proton leak are reported in the left and right upper panel, respectively. The ECAR was measured after Seahorse XFp Glycolysis Stress Test, and resulting data calculated for Glycolytic capacity, Glycolytic reserve, Glycolysis and Non-Glycolytic Acidification are reported in the left and right lower panel, respectively. Columns, mean values obtained from three different independent experiments, bars, SEM. *Significant difference ($P<0.05$) between the two cell lines.

Figure S2: Hypoxia affects pemetrexed activity. Cell growth inhibition was performed in cells exposed to pemetrexed 10 µM PMX (upper panel) or 0.1 µM PMX (lower panel) for 72 hours under hypoxic vs. normoxic conditions, as compared to drug-free control cells. Columns, mean values obtained from three independent experiments; bars, SEM. *Significantly different ($P<0.05$) when compared to the same treatments under normoxic conditions.

Figure S3. Interaction between PCFT and LDH-A. (A) Protein network visualization from the STRING website. The online database STRING (http://string-db.org), which allows retrieving the functional and physical interactions of proteins, did not reveal a direct physical interaction between PCFT and LDH-A. **(B)** Proposed hypothetical model of PCFT and LDH-A modulation in cells growing in hypoxia and as spheroids and after targeted downregulation of PCFT expression.

Figure S4. Interaction between chemotherapeutic drugs and LDH-A inhibitors. (A) Apoptosis induction by 1 µM NHI-2 and 1 µM pemetrexed (PMX) and their combination for 24 hours pemetrexed in H2452 cells growing as monolayer under hypoxic conditions. The apoptotic index was calculated after bisbenzimide-HCl staining, as described previously [Massihnia et al., J Hematol Oncol 2017]. **(B)** Cell growth inhibition by NHI-Glc-2 alone, and in combination with gemcitabine at IC_{25}. Growth inhibition curves of MesoII cells of a representative experiment with NHI-Glc-2 alone, and in combination with gemcitabine, under hypoxic conditions. *Points*, mean values obtained from three independent experiments; *bars*, SEM.

Figure S5. Increased LDH-A mRNA levels in spheroids. Modulation of LDH-A expression as assessed in MesoII and STO spheroids by qRT-PCR. *Columns*, mean

values obtained from three independent experiments (the SEM values were not reported since they were always below 5%).

Figure S6. Scheme describing the analysis of spheroid aggregation. To detect the light passing through the spheroids, pixel intensities of 8-bit black/white-converted images were calculated using ImageJ Software (U.S. National Institutes of Health, Bethesda, MD, USA), as described in the supplemental methods.

Figure S7. Microarray data analyses showing higher LDHAL6A expression in DMPM tissues compared to normal mesothelium tissues. (A) Differential analysis of mRNA expression of LDHA, LDHAL6A, LDHB in Mesothelioma and Normal tissues. LDHAL6A, a protein belonging to the LDHA family, is significantly overexpressed in mesothelioma tissues in the GSE112154 dataset. **(B)** Analysis of shared processes between LDHA and LDHAL6A.

Figure S8. Modulation of tumor volume in orthotopic DMPM tumors. Upper panel: Representative BLI images of mice harbouring orthotopic DMPM tumors. At the start of the experiment, mice were stratified into groups with comparable BLI signal, and then treated with NHI-GLC-2 administered i.p. for 2 weeks. Lower panel: Tumor growth as detected by BLI analysis. *Columns*, mean values obtained from the measurements in three mice; *bars*, SD.

Figure S9. Body weight of animals inoculated subcutaneously with DMPM cells. Median weight loss was always <10%, without toxic deaths. These results support the conclusion that the gemcitabine/NHI-Glc-2 combination displays a safe antitumor activity against *in vivo* models of DMPM.

Figure S10. LDH-A immunohistochemical analyses of MPM tissue microarrays (TMAs). Representative TMA cores in the cohorts of MPM patients, illustrating cases with high (upper panels) and low (lower panels) LDH-A expression (at 4X, 10X and 40X original magnification). Of note, at 40X the LDH-A staining seems membrane-bound. However, this is an artefact. As clearly detected at the other magnification the staining is indeed cytoplasmatic. As clearly detected at the other magnifications the staining is indeed cytoplasmatic. Since these cells have very big nuclei, these nuclei are taking most of the space in the cytoplasm and the staining seems located near to the membrane at 40X. However, this staining is not a “membrane staining”, as clearly demonstrated also by the comparison to our previous staining of EGFR in MPM tissues [Giovannetti et al., Br J Cancer 2011].

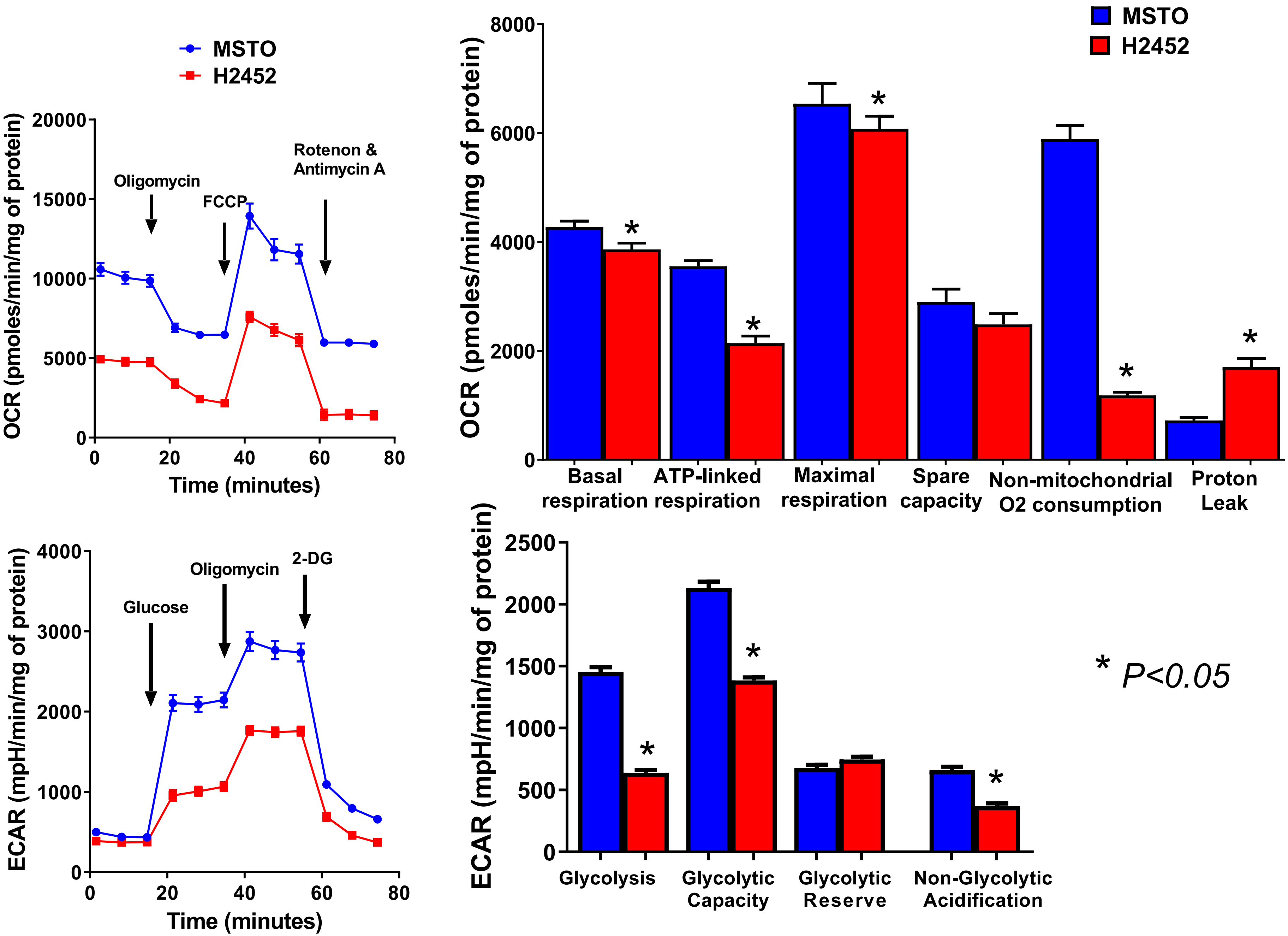

Supplemental Figure 1

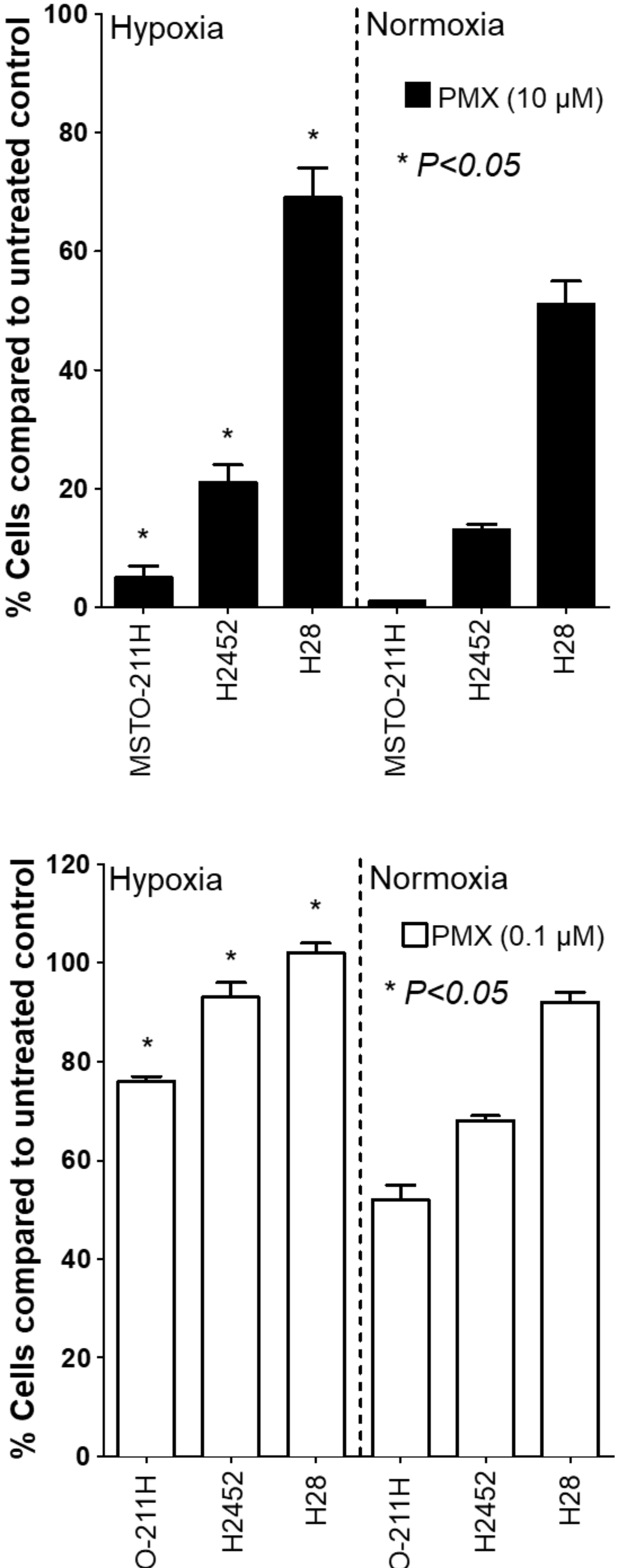

Supplemental Figure 2

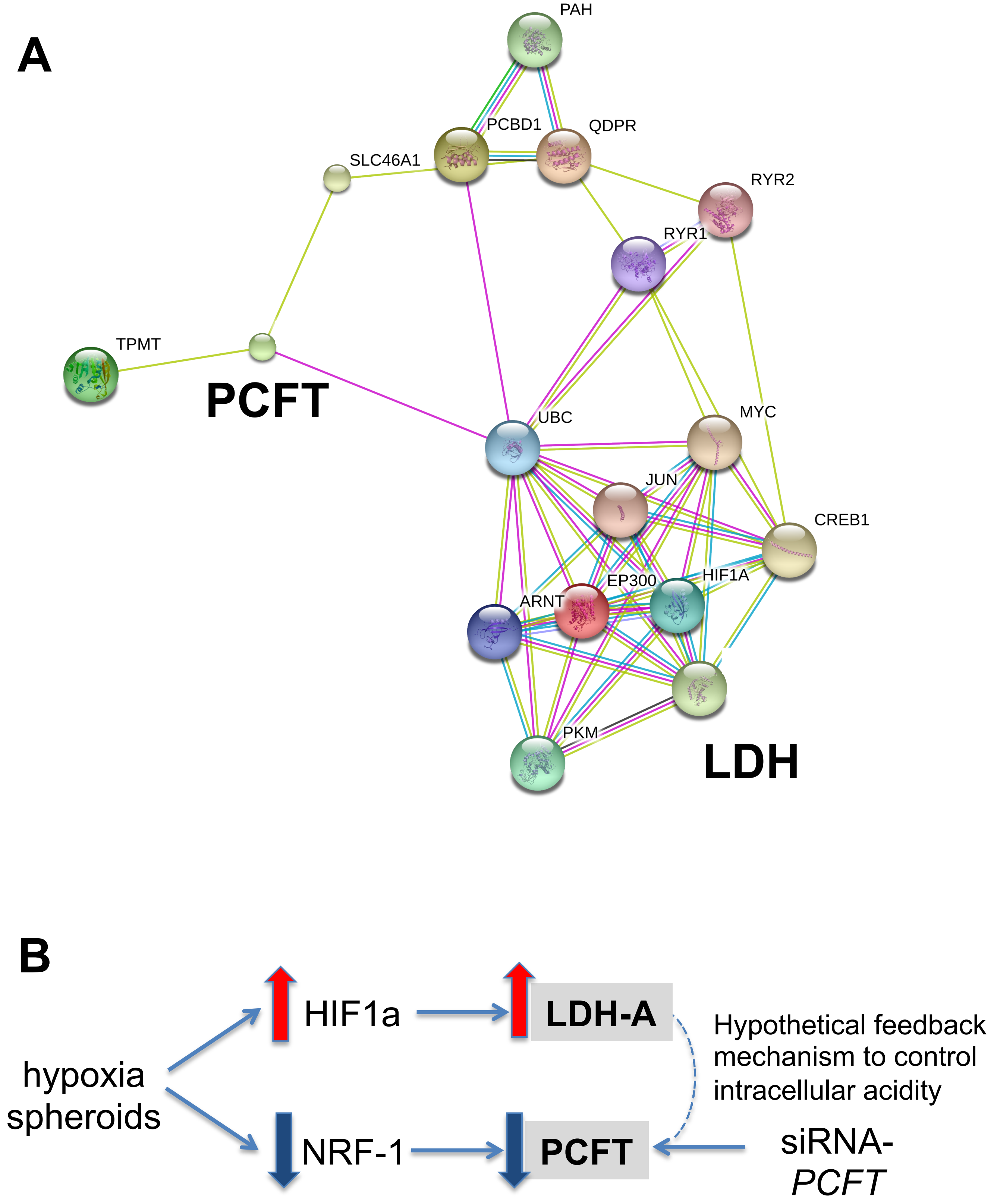

Supplemental Figure 3

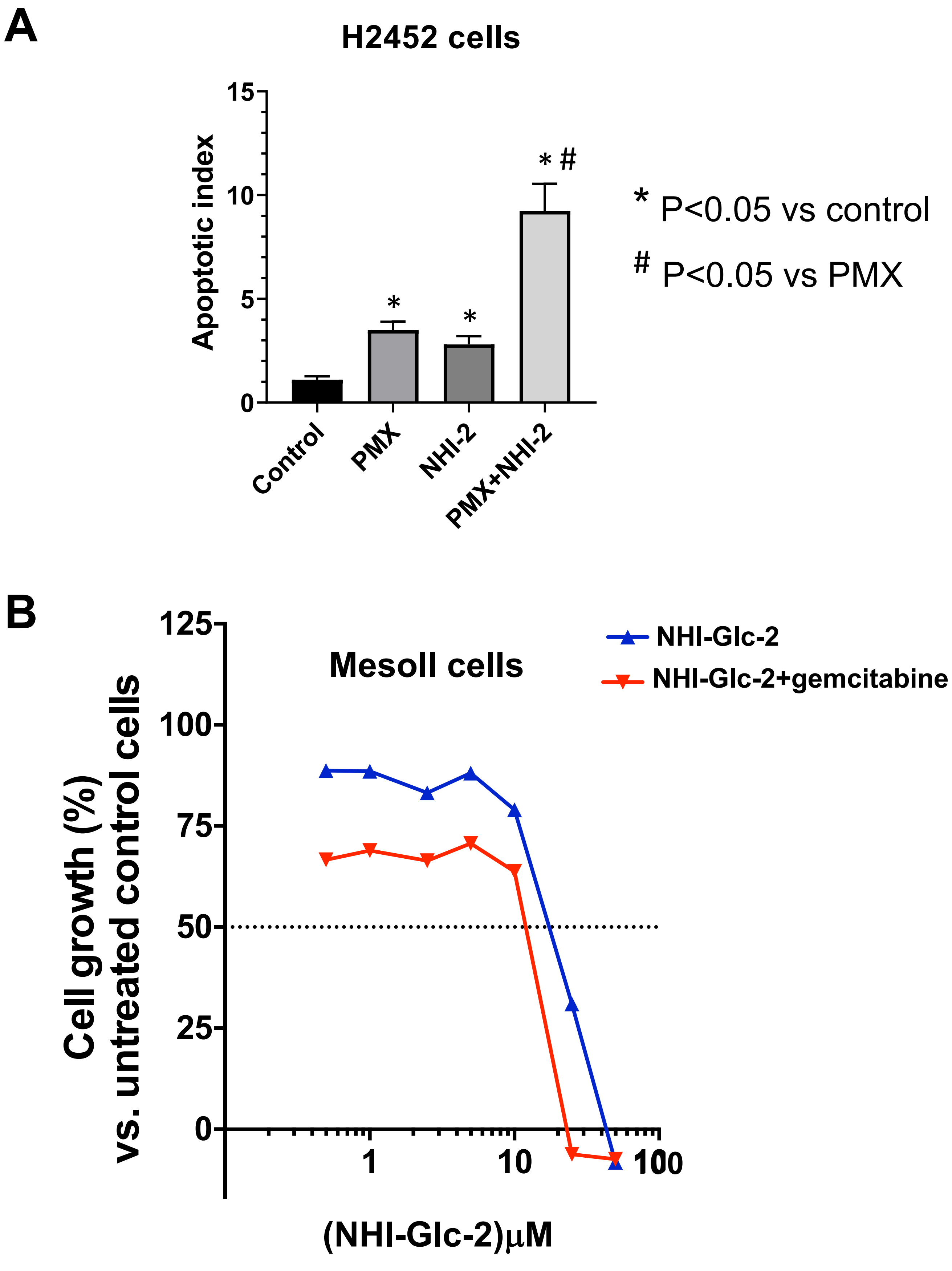

Supplemental Figure 4

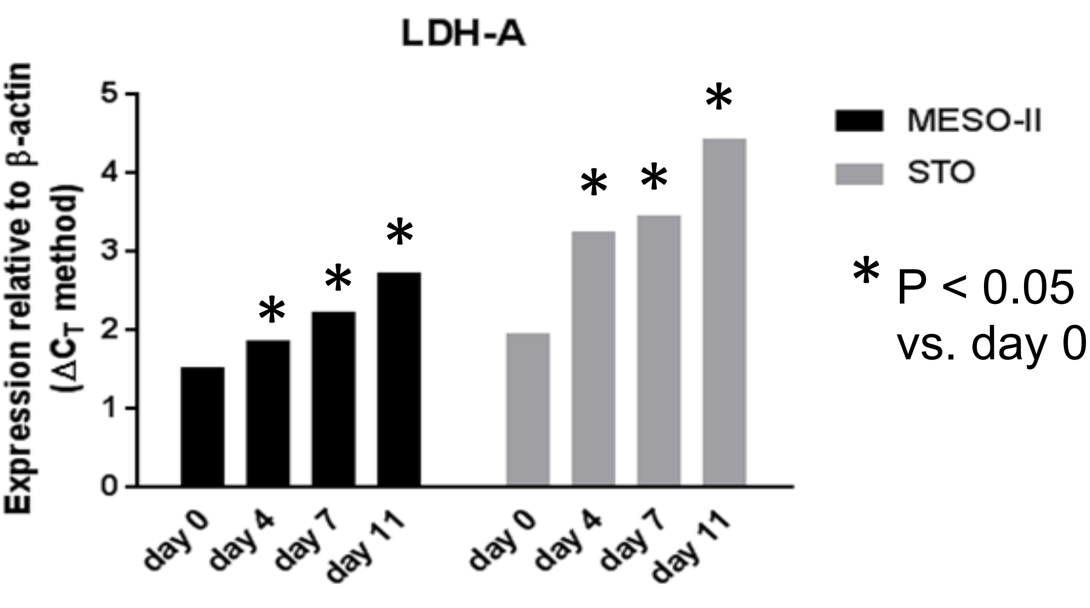

Supplemental Figure 5

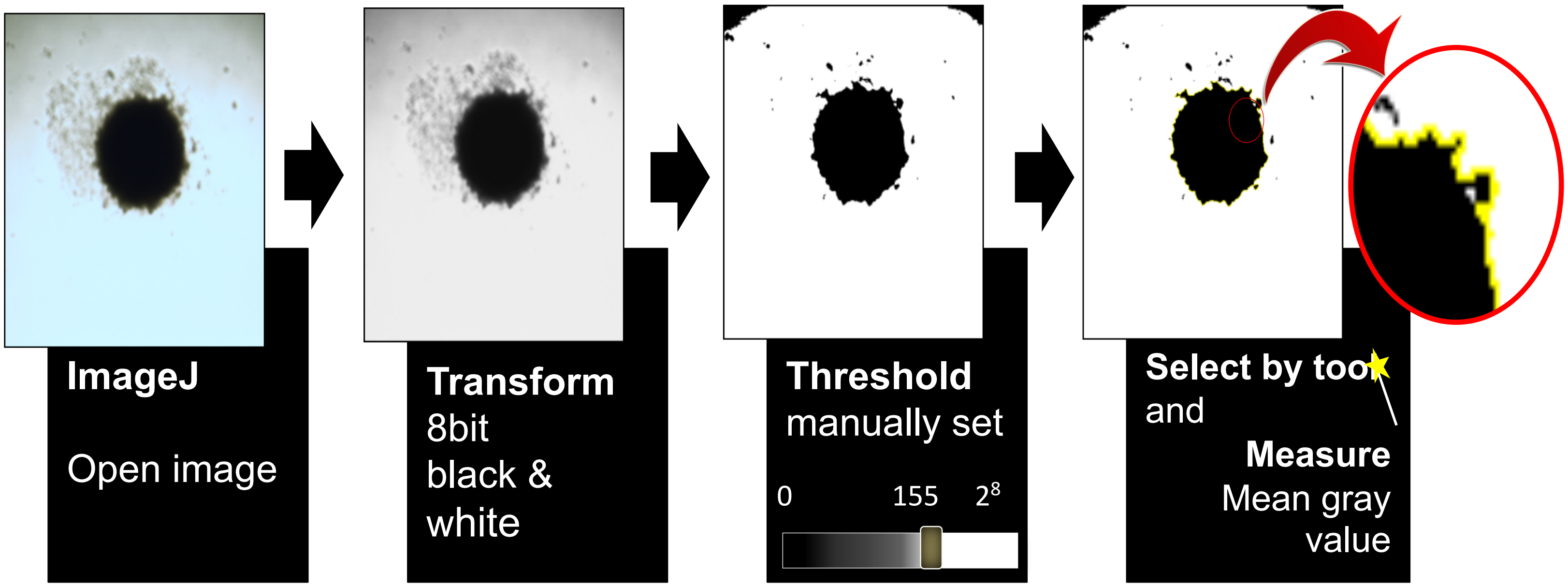

Supplemental Figure 6

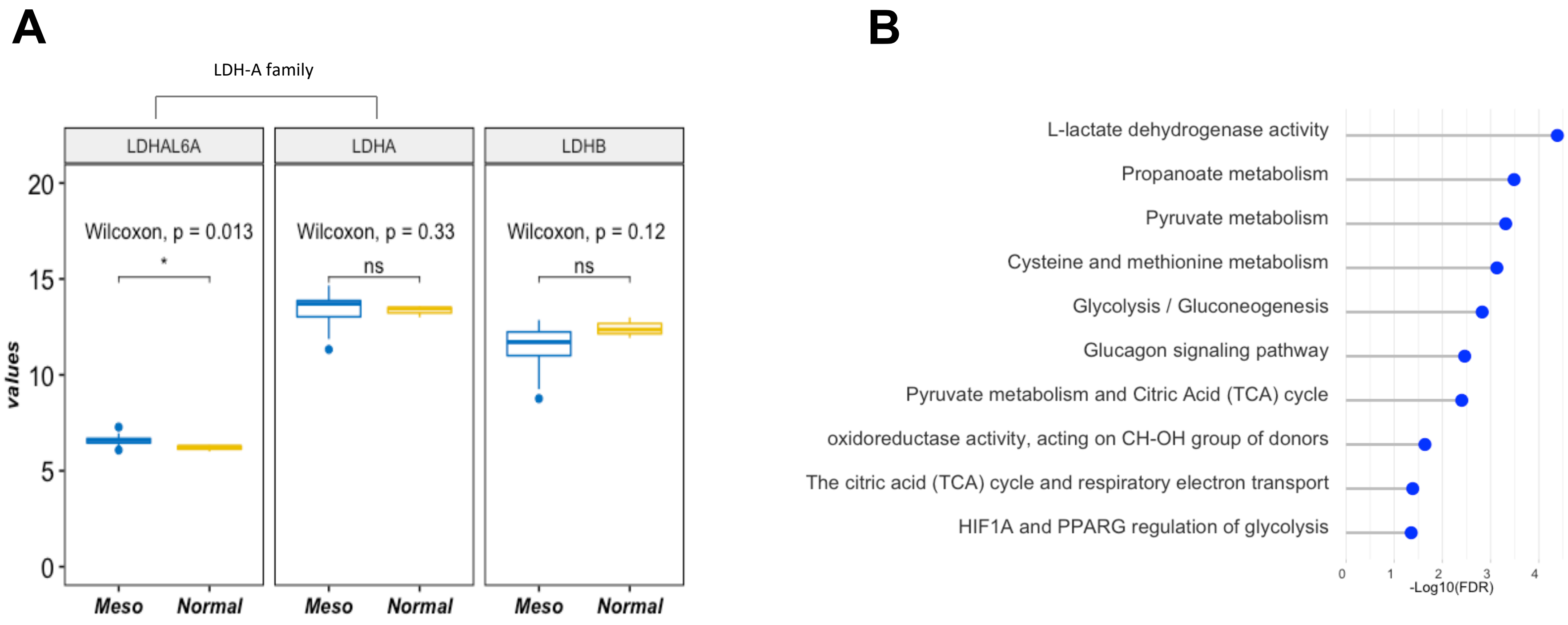

Supplemental Figure 7

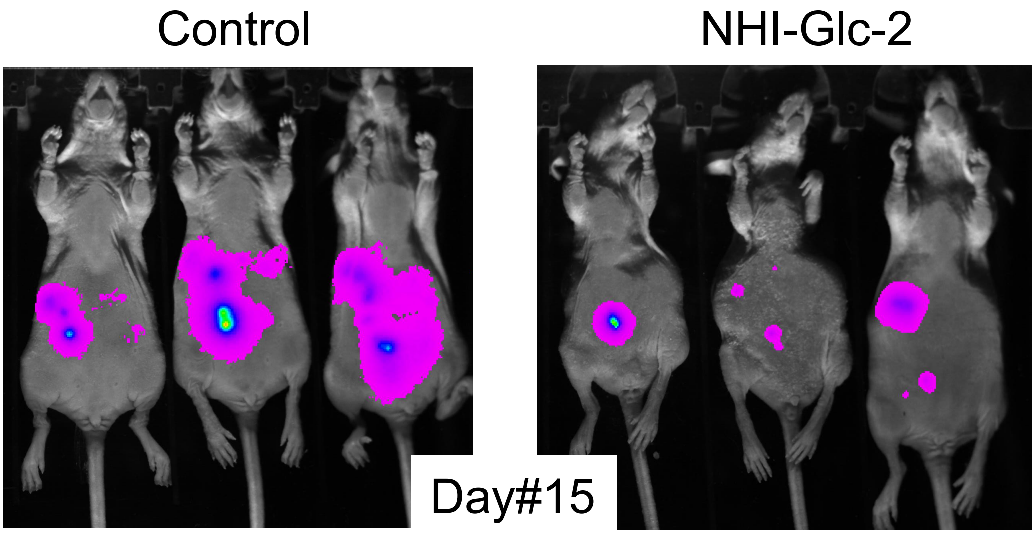

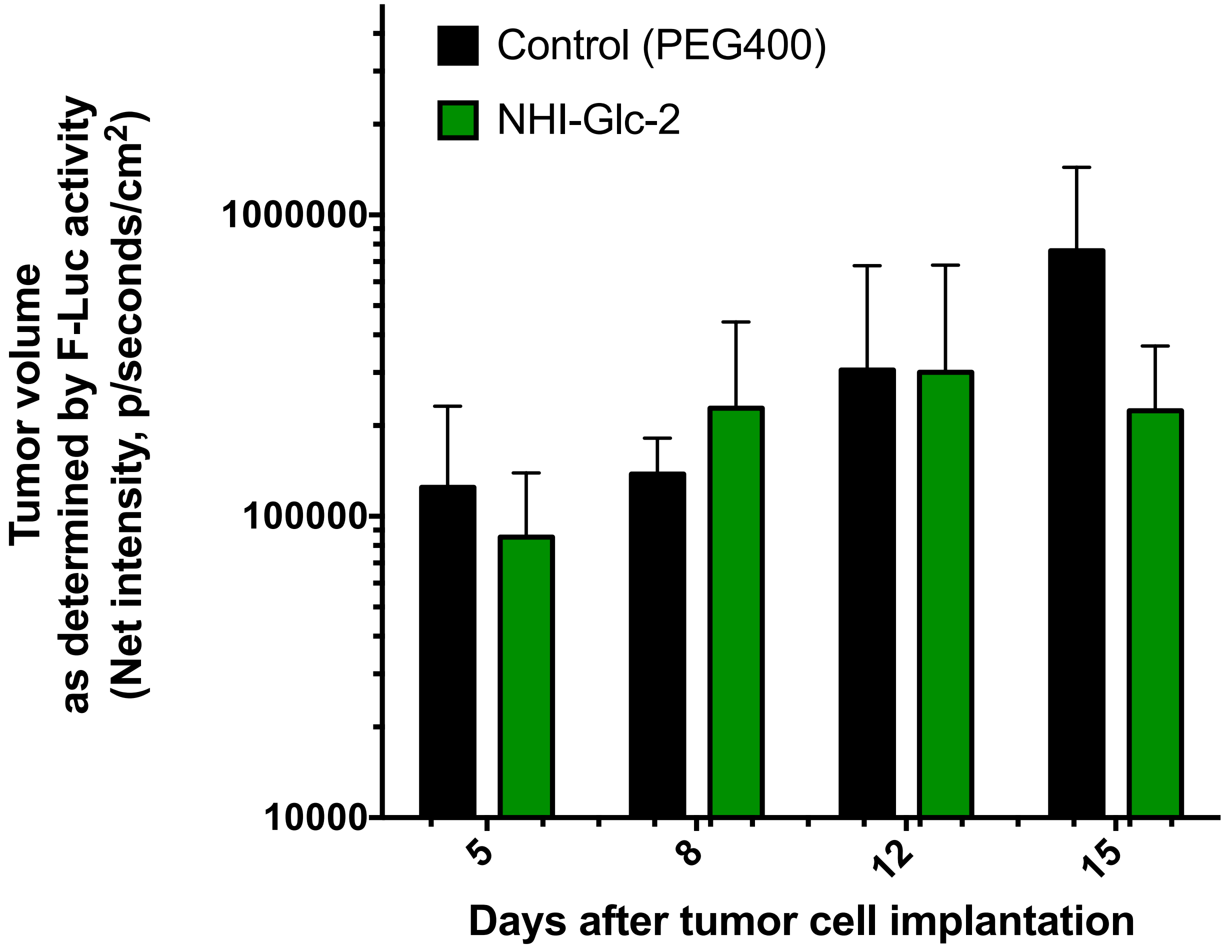

Supplemental Figure 8

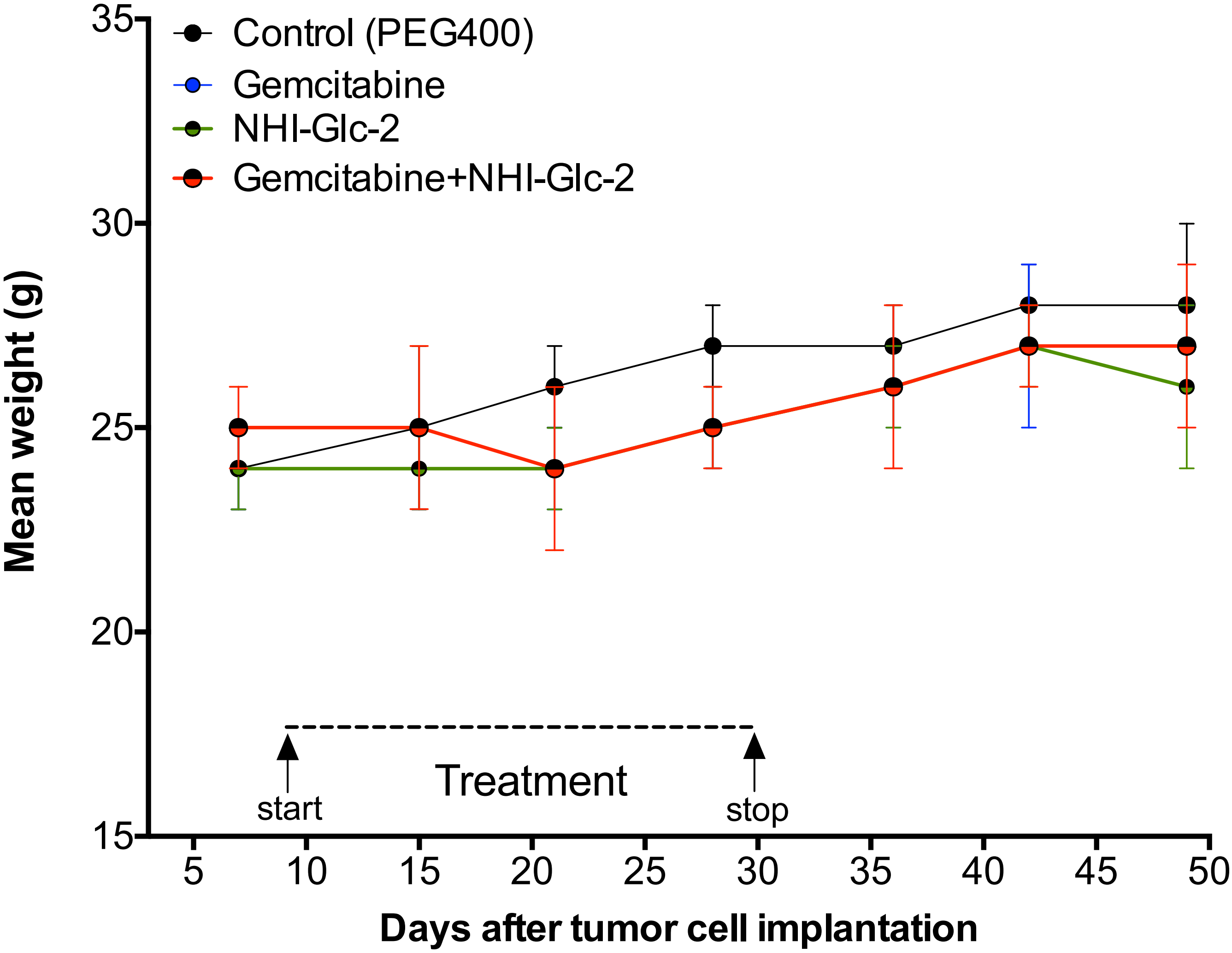

Supplemental Figure 9

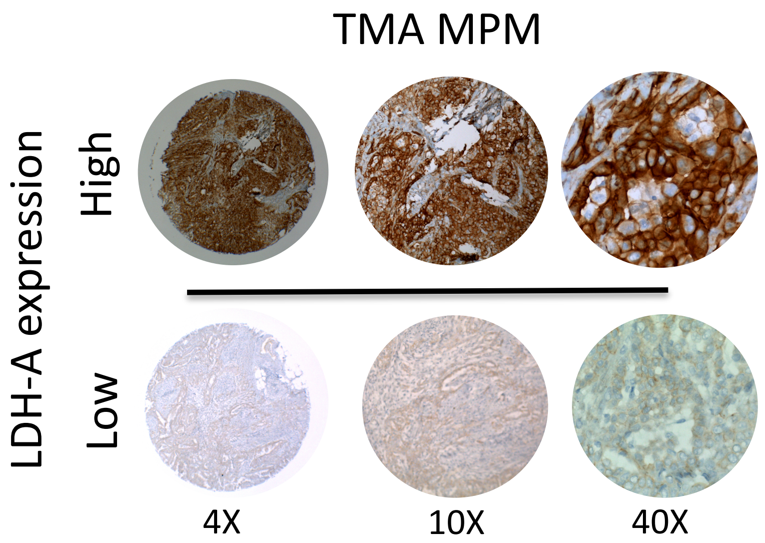

Supplemental Figure 10
